# Renaming the ‘OS-D/CSP’ Family (Part 1): ‘4-Cysteine Soluble Proteins’ (4CSPs)—Molecular Nomenclature, Structure, Expression, Evolution, Tissue-Distribution, and Pleiotropy

**DOI:** 10.3390/insects17020202

**Published:** 2026-02-13

**Authors:** Guoxia Liu, Botong Sun, Wei Fan, Shousong Yue, Qiuxia He, Jean-François Picimbon

**Affiliations:** 1 Shandong Academy of Agricultural Sciences, Jinan 250100, China; girlgx@sina.com (G.L.); yueshousong@163.com (S.Y.); 2 Science and Technology Service Platform of Shandong Academy of Sciences, Shandong Academy of Sciences Foreign Students Pioneer Park, Shandong Academy of Sciences, Jinan 250103, China; sunnybotong@163.com (B.S.); fanwei0928@163.com (W.F.); heqx@sdas.org (Q.H.); 3 Shandong Institute for Quality Inspection, Jinan 250100, China

**Keywords:** 4CSP, 4-Cysteine Soluble Protein, protein evolution, gene structure, ubiquitous expression, immune response, insecticide resistance, adaptation

## Abstract

Chemosensory proteins (CSPs) are found in the olfactory sensory organs (antennae and maxillary palps) and/or gustatory sensory organs (labellum and legs) and have long been accepted to function through the binding of odorants. However, the same CSPs are also expressed in many tissues other than olfactory and gustatory organs, such as the gut, brain, fat body, wing, epidermis, *Corpora allata*, salivary gland, pheromone gland, prothoracic gland, etc. In this report, we suggest renaming the “chemosensory protein (CSP)” the “4-Cysteine Soluble Protein (4CSP)”. This paradigm and nomenclature shift is based on molecular characteristics, genomic mining, tissue distribution, and functional roles beyond those related to olfaction. We examined prior studies on this protein gene family to bolster the renaming, highlighting the most recent findings that we ascribe to “pleiotropic properties” and evolutionary relevance rather than smell. The scope of the report, per se, is broad, and this is especially true given the volume of data that has been gathered on 4CSP expressed in ways that are not consistent with the olfactory paradigm. Statements outlining the many chemosensory properties of 4CSPs, particularly how they activate olfactory receptor neurons (ORNs), are currently scarce, if they exist at all. Many debates currently focus on 4CSPs’ non-chemosensory functions, which are backed by a multitude of evidence, from gene evolution to tissue distribution. Therefore, strong arguments in favor of renaming chemosensory proteins are becoming evident here, outweighing the drawbacks.

## Simple Summary

<<Chemosensory proteins (CSPs) are small soluble proteins that mediate olfactory recognition at the periphery of sensory receptors in insects, similarly to odorant-binding proteins (OBPs)>>?. We argue that the nomenclature of this insect protein superfamily is outdated (it is based on early studies on its presence in the aqueous lymph of antennal sensilla). However, given the collective sum of research conducted on this topic over the past 20 years, the new name for the protein molecule superfamily needs to be far more accurate. We suggest changing the name of this protein family to reflect the most recent findings about its locations, roles, and characteristics. We suggest changing the protein’s name from “CSP” to “4CSP” (4-Cysteine Soluble Protein) due to the highly conserved molecular feature of its four neighboring cysteine residues. Then, we suggest using the common peptide five-letter code (“Bommo-4CSP”) for abbreviations of other insect, crustacean, and bacterial proteins.

## 1. Introduction

A few years ago, there was a case regarding the re-evaluation and/or renaming of allatostatins due to their pleiotropic properties, which go beyond their inhibition of juvenile hormone (JH) biosynthesis in the *Corpora allata* (CA) [1]. Another example of an invertebrate peptide family being named for a highly disputed function (such as chemosensing) is insect “chemosensory proteins”. However, a number of state-of-the-art studies and new findings about evolution, molecular expression, molecular localization, and binding properties, along with another 20 years of documented research, strongly suggest different functions that extend well beyond insect olfaction.

Odorant-Binding Proteins (OBPs) and Chemosensory Proteins (CSPs) constitute two distinct classes of small soluble proteins that have historically been grouped under the conventional umbrella of “Odor-Binding Proteins”. Their cysteine patterns—four conserved cysteines in two distinct disulfide bridges for CSPs and six conserved cysteines in three interlocking disulfide bridges for OBPs—are what set these two groups of enigmatic proteins apart. Instead of antenna-specificity, which becomes very questionable, what makes them attractive is their presence in several tissues, at different stages of the insect’s development, and in the gut (and fat body) in response to insecticide treatment. The goal of this review is to revise this established convention, at least for CSPs or OS-Ds (“Olfactory- Specific type D”), as one of the multiple names given to this protein family.

**Figure 1:**
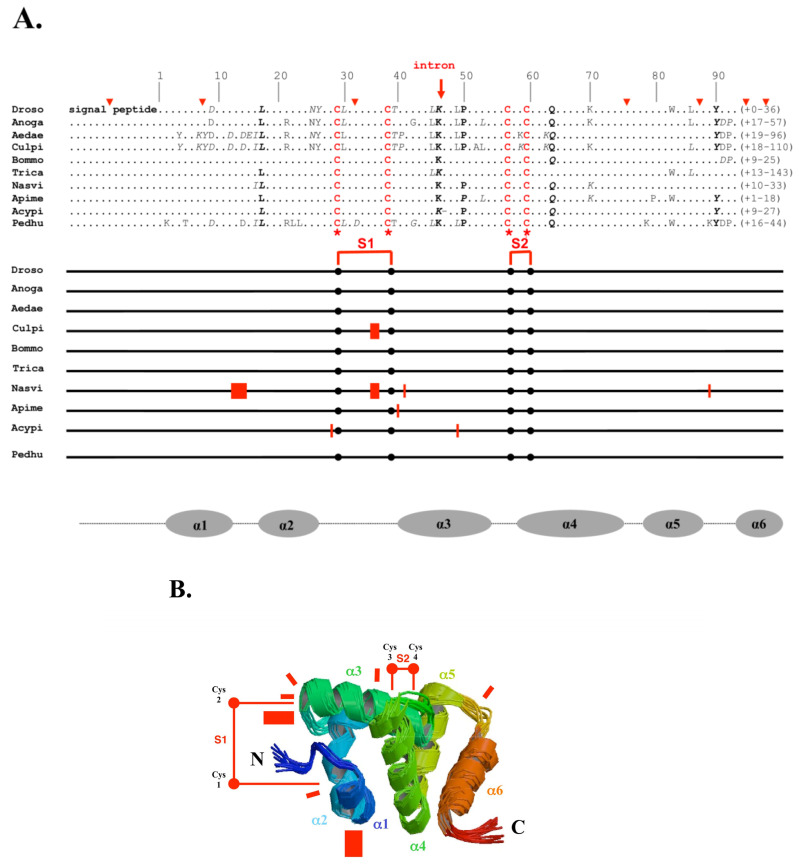
Consensus amino acid sequence alignment and functional structure of insect 4CSPs. (**A**) Comparative study of primary sequences. Twelve *Drosophila* species are referred as to “Droso”: *D. ananassae*, *D. erecta*, *D. grimshawi*, *D. melanogaster*, *D. mojavensis*, *D. persimilis*, *D. pseudoobscura*, *D. sechellia*, *D. simulans*, *D. virilis*, *D. yakuba*, and *D. willistoni*; Anoga: *Anopheles gambiae*; Aedae: *Aedes aegypti;* Culpi: *Culex pipiens*; Bommo: *Bombyx mori*; Trica: *Tribolium castaneum*; Nasvi: *Nasonia vitripennis;* Apime: *Apis mellifera*; Acypi: *Acyrthosiphon pisum*; Pedhu: *Pediculus humanus humanus*. The amino acids shown in bold are shared by the vast majority of 4CSPs. Each dash represents an amino acid. The red and bolded amino acids are those that are strictly preserved in all 4CSP sequences. 4CSPs are characterized by four cysteine residues (4-C), which are shown by the red stars in their respective locations. When comparing 4CSPs from the same species, amino acid residues that change in the 4CSP sequence are indicated by italicization. With Edman degradation [3,15], the first amino acid in the N-terminal sequence and signal peptide of insect 4CSPs are identified. Between Cys29-Cys37 and Cys56-Cys59, the squares (in red) precisely represent the locations of point mutations. Sizes of the squares vary. This corresponds with a certain amount of amino acids (from one amino acid to a full block or motif of amino acids). The *4CSP* genes’ introns are consistently found after Lysine 45 (see red down arrow). The intron fragments the codon for amino acid 46 (Glu, Ser, Lys, Asn, or Asp). The red triangles indicate the locations of insertion for additional introns. Gray circles beneath the alignment indicate the location of functional elements (α-helices α1–α6). (**B**) 4CSP’s 3D structure (2JNT). The locations of point mutations on the CSP 3D structure are indicated by the red squares. The disulfide bridges (S1 and S2) and interlocked cysteines (Cys1-Cys2 and Cys3-Cys4) are shown in red. The main site of mutation is around Cys2.

“CSPs/OS-Ds” are thought to mediate the recognition and transport of chemosensory molecules to olfactory receptors (ORs) at the periphery of sensory dendrites in the insect *sensillum* [2,3,4,5,6]. According to Lartigue et al. [7], CSPs are composed of six α-helices with an approximate molecular weight of 10–12 kDa (or 110–120 amino acid residues); four cysteines that form two nearby disulfide bridges, which each connect two closely spaced cysteines; and a narrow functional hydrophobic tunnel structure (see Figure 1). So far, four CSP structures have been determined in locusts (*Schistocerca gregaria*) and moths (*Mamestra brassicae*, *Bombyx mori,* and *Spodoptera litura*) [7,8,9,10,11]. Jansen et al. described CSP as a “prism” that is well suited for interacting with a long-chain fatty acid (FA) [8,9]. The ligand-binding site is flexible enough to accommodate various ligand sizes [12,13]. The hydrophobic tunnel of CSPs’ affinity for long-chain lipids and structural, molecular, or biochemical interactions both point to a function in lipoid transport, which is essential in many different regulatory systems. The tissue distribution and developmental profile of this protein family are well matched to its biological role in numerous regulatory systems. This is the first compelling argument we will make in Part 1 (this paper) to refute the term “chemosensory proteins” to address the entire protein gene family; Part 2 is the second half of the review concerning renaming based on the intracellular functions of CSPs [14].

## 2. CSP: An Example of Research-Study Spin or Interpretation Bias

“Spin” refers to general reporting practices that present study results in a more positive light than the data warrant. An unwarranted and disproportionate sense of optimism regarding the performance of proteins may result from the practice of “spin”, which involves distortion and overinterpretation. In clinical trials and biomedical literature, spin is quite prevalent, primarily in the identification of disease biomarkers. It affects how newcomers interpret study findings or the state of research, which may get very complicated if we discuss gut, CA, and ovarian biomarkers for odor. Much like the biomedical literature at risk, “CSP” research could be easily incorporated into “spin” by assessing these proteins’ ability to smell too quickly and relying too heavily on what is still a supposition. The strong expression of the antennae seen by numerous authors, particularly in earlier works [2,3,4], was likely sufficient to guide the study of this protein in the area of chemosensing. Quantitative real-time PCR, Illumina sequencing, transcriptome libraries, and genomic databases were not available for early works. A role tailored to chemosensing, however, is obviously refuted by the most recent findings, and future research should seriously consider this issue.

CSP molecules are present in insects at all steps of their life cycles, from egg and larva phases through immaturity and adulthood [15,16,17,18]. In locusts (*Locusta migratoria*, Locmi), CSPs are mostly expressed in the legs and antennae of both sexes, and they have been linked to phase shift (phenotypic plasticity) in that species [19,20,21]. Nevertheless, all CSPs in moths are upregulated in most insect body tissues after exposure to avermectin pesticide molecules, particularly in the gut and fat body [22]. In contrast to a role in olfaction, this is important for emphasizing a role in tissue growth, development, and pesticide resistance. The problem is that, although it is well known that *CSP* expression occurs in non-sensory organs and during the early stages of insect development, research on the functional roles of these proteins has been largely and stubbornly conducted using a set of semio-chemicals, recombinant protein testing, and competitive fluorescent binding experiments as described by Ban et al. [23]. Fluorescence displacements are often used to assess the function of these proteins, and the identity and purity of the protein are often contentious concerns. Most of the binding experiments that have been reported in the functional characterization of CSPs used the 1-N-phenylnaphthylamine (NPN) binding assay. It is known that this method has significant limitations that increase the likelihood of false positives or false negatives [24]. First off, NPN is a hydrophobic fluorescent probe that can bind to any hydrophobic location on or within the protein structure, without any specificity. Second, due to variations in the binding site’s shape and sequence, CSPs from different species may have varying affinities for NPN. This is shown by the varied values of the displacement constant (*Ki*) when the ‘first ligand’ is NPN. Third, the additional odorant (‘second ligand’) in the competitive binding experiment has the ability to build micelles and trap the probe, increasing the observed fluorescence without exhibiting any functional outcomes. Although a lower *Ki* value indicates that the titrated ligand displaces the NPN probe extremely effectively, this does not imply that the ligand will bind strongly at equilibrium [24]. Therefore, it seems that this NPN-based method, which is widely used to describe the functional properties of CSPs, is not the most accurate way to determine equilibrium constants. NPN can likely be used as a screening method to investigate displacement efficiency with different ligands, provided that the list of tested ligands is very large [25]. Based solely on the evaluation of this chemical, it is completely irrelevant to establish a link between CSP and the binding of an odorant.

Furthermore, it must be made very clear whether the original protein or an isoform variant is expressed in the recombinant system. The number of loci and genomic organization are frequently overlooked in this research. The number of mRNA transcripts is frequently utilized and mistakenly understood as the number of genes. An mRNA clone should not be referred to as “a CSP gene” when studying a gene family like *CSPs*; instead, genomic DNA analysis that considers the number of loci and genomic organization is necessary. This distinction between transcript and gene is important for *CSPs*, for which RNA and protein editing has been shown to be effective [26,27].

Numerous studies, such as those by Sun H. et al. [28], Khuhro, S.A. et al. [29], Li, Z. et al. [30], Yang, Y. et al. [31], Yin, M.Z. et al. [32], Liu, Q. et al. [33], Li, J.B. et al. [34], Jiang, J. et al. [35], and Zhao, S.H. et al. [36], claim that CSPs have a direct role in olfactory perception. Not all of the evidence is convincing. Concerns that remain important include the biochemistry work’s relevance, the protein sample’s level of purity and integrity—and sometimes even the protein’s identity—the protein’s folding, the binding assay (compound interference and non-specific gross structural changes to the protein, which can lead to a large number of false positives in fluorescent spectroscopy), the work’s repeatability, and the “antennae-specificity”, which is questionable because it has never been really examined [28,29,30,31,32]. The role of *Holotrichia oblita* Falderman’s CSPs was not entirely solved by Sun et al. [28]. Holob CSP1 and CSP2 were not pure, and there were few binding trials, only one binding value, no replication, and no information on tissue distribution [28]. Similar to the labeling of *Schistocerca* with the same type of antibody, the labeling of the sensilla with (polyclonal) antibodies was quite diffuse [4,28]. We can also consult the *Chilo suppressalis* (Chisu, Crambidae) experiment for all these issues [29]. The major finding of this experiment is that the olfaction perception of plant semio-chemicals is directly influenced by these CSPs. This is not very convincing, particularly when considering the degree of purity of the protein samples in the SDS gels (which have numerous protein bands, only one of which should be examined in a binding assay) and the method used to “measure” antennae-specificity. For biochemical work, highly pure protein samples would yield a more accurate and relevant analysis. Therefore, it would have been much more acceptable to test an equal number of tissue samples rather than 200 antennae, as opposed to just 20–40 heads and abdomens, in order to validate antennae-specificity (see [29,30,31,32]). Because the protein sample is not pure from the start, including numerous impurities of varying sizes, and the CSP is biased to antennal expression, comparisons of its relative binding—also utilizing only a few odors or semio-chemicals—cannot be used to make conclusions [29,30,31,32]. Only one tissue (the antennae) and a small number of chemicals (pheromones) were examined for *Riptortis pedestrus* [32]. Yin et al. claim that the binding of aggregation pheromone (*E*)-2-hexenyl (*Z*)-3-hexenoate is responsible for the variation in CSPs with age [32]; however, this is questionable because the aggregation pheromone in Rippe varies with diet and focuses on tetradecyl isobutyrate [37]. Liu Q. et al. and Li J.B. et al. made the same mistake (distortion: antennae-biased and no tissue-distribution analysis) [33,34]. Strangely, Yang et al. identified gnat CSP as an olfactory gene for sulfur compounds while finding higher expression in the head (in the brain, no ORNs) [31]. This is an example of overinterpretation because the precise expression of the gene was not carefully taken into account. Furthermore, because CSP binds a ligand that influences insect behavioral responses, it is not very rigorous to say that it is olfactory (see [31]). It is nonsensical to consider a liking for a sex pheromone or a plant odor if the gene is expressed in the brain or the gut. According to Xuan et al. [22], CSPs primarily target the gut and fat body, and the abdomen should be carefully examined rather than merely employed as a control [35]. A transcriptome of the antennae does not necessarily indicate antennae-specificity, too, if it is not compared to transcriptomes of every other tissue. Without providing any functional characterization, Zhao et al. merely discussed antennal transcriptomics and related CSPs with ORs based exclusively on their expression in the antennae [36]. The first issue of distortion and overinterpretation is the claim that the gene is olfactory because it is expressed in the antennae. When such a study is acknowledged and/or viewed as a potent illustration of CSP function, it becomes rather difficult to solve the CSP puzzle thirty years later. There is an olfactory bias and spin in CSP research.

Speaking more generally about olfactory studies on CSPs, it seems that the ligand-binding experiment was somewhat biased toward ‘olfactory’ ligands, which presents yet another significant issue in CSP research. The biological relevance of the odor ligand molecules under test for a CSP identified in the stomach and fat body is often unclear, especially when examining behaviors, host-plant volatiles, and pheromones. It is hardly ever correlated with a ‘logical’ ligand or one that could be bound by a protein that is so broadly expressed in the ovaries, eggs, legs, antennae, wings, thoracic glands, fat bodies, and hindguts. Surprisingly, despite the fact that such expression profiling would focus research on hormone or lipoid transport rather than olfactory molecules, the overall approach leaves out lipoids for sex pheromones and plant semio-chemicals. The exact tissue distribution of the protein has either never been thoroughly investigated or not even considered while choosing a ligand molecule for binding tests. Experiment have often been conducted randomly and without any kind of rationale, such as by testing a chemical that is relevant to insect biology. Consequently, the protein’s functional importance remains controversial, particularly when binding studies only compare randomly chosen scents or fail to examine a broad range of compounds.

Oleamide is one such instance that has been documented for being a potent inhibitor of the branching morphogenesis of the salivary mandibular gland, as well as a putative “CSP” ligand (see [23]). If it is related to defense and development rather than smell, binding to CSPs makes sense. One key element in identifying the true physiological ligand should be the location and timing of the protein molecule’s expression. OBPs from *Aedes aegypti* (Aedae) mosquitoes are also subject to similar misinterpretations and/or overinterpretations (spin). These OBPs are expressed throughout the insect body, including in the antennae, legs, and abdomen, but are only partially characterized by a limited number of randomly chosen semio-chemicals [38]. Since this molecular analysis did not examine any of the odors found in Aedae from eggs, pupae, nymphs, and adults, in various tissues, there is much uncertainty regarding OBPs’ functions [39]. For instance, testing should be done on the methyl tetradecanoate molecule (C_15_H_30_O_2_), which is highly prevalent in the female thorax. Testing should then be done on chemicals such as (*Z*)-9-hexadecenoic acid methyl ester (C_17_H_32_O_2_), hexadecanoic acid methyl ester (C_17_H_34_O_2_), (*Z,Z,Z*)-9,12,15-octadecatrienoic acid methyl ester (C_19_H_32_O_2_), 8,11-octadecadienoic acid methyl ester (C_19_H_34_O_2_), methyl stearate (C_19_H_38_O_2_), and ethyl oleate (C_20_H_38_O_2_) due to their high concentrations in female wing tissue and male antennae [39]. This would provide greater proof of OBPs’ functions in pheromone binding. These two instances show how elusive and unclear the ligands—and, thus, the functions—of CSPs and OBPs are at this time.

Like OBPs, it is now commonly recognized that following insecticide poisoning, microbial infection, or viral infection, an increased load of “CSPs” is observed fly hemolymph and in all insect tissues [22,40,41,42,43]. Upon identifying that these CSPs were overexpressed in fly hemolymphs following bacterial or viral infection, they were renamed “pherokines” in an early attempt to disassociate these proteins from odors.

Transcriptome datasets gathered from different insect species that have analyzed treated and untreated samples in regard to the three mentioned challenges (chemical, microbial, and viral) can further illustrate the role in relation to the immune system [44,45,46,47,48,49,50,51]. In the majority of these studies, “*CSPs*” and “*OBPs*” have been identified as differentially expressed genes according to chemical toxicity or microbial/viral assault. Therefore, both families exhibit a connection to the immune system, though not necessarily through olfaction.

In order to gain a better understanding of the immune response’s mechanism of action, Liu G. et al. used the sweetpotato whitefly (*Bemisia tabaci* (Bemta)) as a model study to address the unique role of CSPs in lipid transport in connection with pesticide resistance (see [41,52,53]). According to Liu et al. [52,53], the “Bemta-CSP1” protein is upregulated by insecticides and interacts with the long-chain C18-fat lipid (C18:2, linoleic acid, LA). Bemta-CSP1 expression rises in a dose-dependent manner in whiteflies treated with neonicotinoid Thiametoxam. However, Bemta-CSP1 has been shown to bind LA instead of thiamethoxam, indicating that CSP participates in the insecticide response through LA and lipid metabolic pathways [52]. This was the first thorough and innovative study that focused “CSP” research on chemical toxicity, insecticide poisoning, lipoid transport, and insecticide resistance rather than olfaction.

Based on these findings, we assert that the protein should be renamed and that this renaming covers not just moths, mosquitoes, and whiteflies but also other insects and perhaps even non-insect taxa like bacteria and crustaceans. Similar proteins from other insect species (such as *Drosophila*’s OBP, which is extensively expressed in tissues other than the gustatory and olfactory sensory organs during all stages of development) should likely also be renamed. However, the research report does not address OBPs or OBP-like proteins; instead, it focuses on “4CSP” and related proteins, for which there is mounting evidence of a role beyond the odor paradigm.

## 3. History of the Discovery of “4CSPs” and Their Pleiotropic Functions

The history of the different names given to this family of proteins, dating back to 1992 with p10 [54], is compiled in Table 1. Between 1994 and 2000, the protein family underwent four name changes, despite the fact that p10 was discovered in *Periplaneta americana* (Peram) leg regeneration tissue rather than antennal sensory tissues (Table 1). *Drosophila melanogaster* (Drome) A10 and OS-D, Drome ejaculatory bulb-specific protein 3 (Ebsp3 or PebIII), moth “SAPs” (“Sensory Appendage Proteins”), and locust “CSPs” are all direct orthologs of p10. All of the name alterations had the same obvious and unmistakable significance (“chemosensory” function, confined to “chemosensory” tuning) and disregarded the first member of the protein family to be documented in the literature (p10) [54]. Liu G. et al.’s findings regarding the lipoid-binding function of CSPs are compatible with p10 found in regenerating legs [52,53,54]. The protein family was regrettably renamed to exclusively reflect sensory appendage and chemosensory functions, excluding any potential developmental or lipoid functions, despite the lack of sufficient evidence [55,56,57,58].

The fourth revision to the p10 protein nomenclature was “CSP”, which maintains a strong emphasis on chemosensory function over regeneration, developmental, neuronal growth, and brain-tissue repair functions. But at this point (2025), five significant findings cast doubt on the possible use of these proteins in chemical detection: (1) their overexpression in the hindgut, fat body, and hemolymph in response to infection; (2) their interaction with lipids over infection; (3) their presence in insects’ ejaculatory bulbs, which are “muscular” male secretory organs; and (4) the injection of aphid Mp10, which activates plant immune systems, and (5) its presence in bacteria and non-sensory tissues of crustaceans [22,42,52,53,57,59,60,61].

There is no proof that “CSPs” interact with sensory neurons; thus, even after more than 20 years of research, their mode of action and/or precise role in chemosensing or olfaction are still up for debate. It is not supported by any evidence that CSPs cling to odors and carry them to ORs (see [2,3,4,5,6,7,8,9,10,11,12,13,14,15,16,17,18,19,20,21,22,28,29,30,31,32,33,34,35,36,42,52,53,59,60,61]).

Since 2003, it has been considered that this “CSP” family is pleiotropic and operates independently of the chemosensory system [6,42]. The term “Pherokine” describes the extremely common “CSP”-like molecules in flies’ hemolymph that result from bacterial or viral infection or contamination with pesticide chemicals ([31,52,53]; see Table 1). This adds “CSPs” to the insect defense system and immune responses, not chemosensing. As a result, a growing body of research has been done on this family of binding proteins, revealing roles that go far beyond the olfactory system, the sensory branches, and the antennal sensilla [14].

What distinguishes A10, SAP, CSP, Mp10, pherokine, OS-D, and p10 from every other protein in the same family? The existing nomenclature (Table 1) completely ignores the most amazing feature of this family of protein molecules, which is expressed throughout the whole insect’s body [22]. To also account for all family members across species of arthropods, crustaceans, insects, isopods, and microbes in the vast bacterial prokaryotic superkingdom, the entire protein family should be renamed “4CSP”.

## 4. Broad Survey and Analysis of 4CSPs in Insects and Hexapods

Examining a protein family that functions in such a diverse range of insects and hexapods begs the question of how species or phenotypes affect the core sequence. This is crucial if we want to preserve the notion that the primary sequence’s evolution is closely linked with a very specific function in the chemosensing of odor and, consequently, evolved in tandem with the variety of pheromones and odorant volatiles present in plant scents.

### 4.1. Molecular Evidence of RNA Editing

In order to understand how a 4CSP’s primary sequence can be altered, it may be necessary to start with the *4CSP* gene editing seen in silkworms, which results in a variety of 4CSPs that are mostly caused by particular differences in the DNA, RNA and peptide molecules. The genesis and evolution of novel organismal phenotypes in moths may be explained by these facts, which include RNA editing, post-translational modification, and mutations in the 4CSPs. Specific motif insertion, amino acid inversion, +Cys, and the addition of a Glycine residue next to a cysteine at particular locations in primary protein sequences are all indications that the Lepidopteran 4CSP family underwent recoding after protein synthesis (i.e., alterations at the epiproteomic level; see [26,27]). The molecular and functional diversity of these mutations has yielded numerous important findings despite the fact that additional X-ray or NMR proof is required. *Bombyx*’s post-translational modification and RNA editing effects on 4CSP activities were examined. For example, the +Gly mutation abolished α-turn in 4CSPs according to modeling structural analysis (against the 100% identity model) [27,62]. Instead of having redundant activities at different developmental stages, 4CSPs exhibit tissue-specific functions based on protein mutations and structural changes. RNA editing appears to be tissue-specific, happening in tissues as diverse as the brain and wings, and it may be controlled by specific physiological and/or environmental factors (see [26,27,62,63]). One compelling hypothesis is that lipoid transport and pheromone production depend on these 4CSP mutations, which are favored in the female moth sex pheromone gland [26,27]. However, these recoding processes appear to represent a ‘universal’ regulation of 4CSP expression in insects, as editing has also been biotype-specifically reported for whitefly 4CSPs [41,52,53]. These modifications, which include post-transcriptional and post-translational changes, RNA editing, and ribosomal peptide editing, may be the cause of the variations in 4CSP primary sequences amongst different insect species. They might not only reflect editing during gene expression but also reflect distinctive evolutionary modifications (Figure 1).

### 4.2. Comparing 4CSP Sequences from Several Taxa

The 4CSP primary sequences from *Acyrthosiphon pisum* (Acypi), *Apis mellifera* (Apime), *Aedes aegypti* (Aedae), *Anopheles gambiae* (Anoga), *Bombyx mori* (Bommo), *Culex pipiens* (Culpi), *Drosophila* (Droso), *Nasonia vitripennis* (Nasvi), *Pediculus humanus humanus* (Pedhu), and *Tribolium castaneum* (Trica) were compared in order to ascertain whether these modifications could lead to the emergence of a new protein function and, consequently, a new insect phenotype (see Figure 1 and Table S1). Bommo-4CSPs and Pedhu-4CSPs had approximately 27–52% identity, whereas Acypi-4CSPs and Pedhu-4CSPs shared approximately 15–52% identity. Therefore, when compared to lice, aphid and moth 4CSP sequences are equally separated. However, when examining the identity score to order subjects, the Acypi-4CSPs differ from those for both Bommo and Pedhu (Table S1). Bommo-4CSPs are closely connected to Lepidoptera (51–98% identity), but Acypi-4CSPs are closely related to Hemiptera (78–96% identity). Pedhu-4CSPs are more closely related to 4CSPs from Coleoptera, Hymenoptera, and Diptera, with 55–68% identity (Table S1). This makes some sense, as both *Bombyx* and *Pediculus* are holometabolous, whereas the pea aphid and Hemiptera are true bugs.

Comparing body lice, pea aphids, and silkworm 4CSPs to their bacterial orthologs, however, revealed significant differences. Picimbon provided a comprehensive and in-depth analysis of the 4CSP sequences of insects and microorganisms [61]. Hemipteran-4CSPs show ~35–75% identity with bacteria, whilst Lepidopterans and *Bombyx* show slightly higher identity (~39–91%). Bacteria and Pedhu-4CSPs are far less closely related (bacteria–Pedhu comparison: only ~42–63% identity; Table S1). Additionally, bacteria more closely associated with hematophagous insects, including lice, are not the same as those associated with herbivorous insects. Pedhu-4CSPs showed high identity with the 4CSPs from bacillales, lysobacterales, and myxococcales, whereas Acypi- and Bommo-4CSPs showed greater identity with the 4CSPs from alteromodales, bacillales, enterobacterales, kitasatosporales, lysobacterales, and myxococcales, among others (see Table S1 [60,61]). It is not totally clear how pheromones and plant smells relate to these comparisons between bacteria and insects. But rather than evolving a new function for a new phenotype, the identity scores between insect and bacterial 4CSPs appear to be suggestive, at least in theory, of a conserved function and phenotype [61].

It is interesting to note that when comparing the N-terminus, Pedhu-4CSPs grouped with Acypi-4CSPs, but when comparing the C-terminus, they grouped with Bommo-4CSPs (see Figure S1). Acypi and Pedhu were more related than Bommo and Pedhu when comparing the whole primary sequences of 4CSPs (see Figure S2A). Acypi005842, Acypi000093, Bommo-4CSP19, Pedhu54410, Pedhu594420, and Acypi03368 made a very distinct orthology grouping (bootstrap value: 82%), and they were also strongly related to 4CSP sequences from *Bacillus amyloliquefasciens* and *Klebsiella pneumoniae*. Instead of asserting that bacterial 4CSPs are the evolutionary progenitors of insect 4CSPs, the “ancestral” function for the group of Bommo-4CSP19 and orthologs, which includes *Bacillus* and *Klebsiella*, is suggested here (see Figure S2B). Regardless of the insect species, the phylogenetic data suggests that these genes maintained their “ancestral” function for 4CSPs rather than evolving a derivative function (Figure S2B). Ancestral state reconstruction, functional characterization of the protein, and additional comparative analysis of the Bommo-4CSP19 group are needed to bolster this evolutionary theory. Studying the Bommo-4CSP19 group is significant because its members do not link 4CSP to smell. More ecological and functional information on Bommo-4CSP19 orthologs in insects and microorganisms is necessary, since *Myzus persicae* Mp10 and Acypi000097 were found to be 100% bootstrap-linked to Bommo-4CSP19 (see Figure S2). This requires functional validation of a common “ancestral” mechanism unrelated to smell, showing highly conserved similarities between insect and bacterial protein sequences, particularly in the Bommo-4CSP19 group (see [14]).

### 4.3. Proof of Relatedness Between 4CSP, Thap1, and PAN-1 Families

Interestingly, both the identity score (Table S1) and the protein tree comparing the whole primary amino acid sequences of 4CSPs from Acypi, Bommo, and Pedhu (Figure S2B) revealed strong connections between Thap1, PAN-1, and many Bommo-4CSPs. Thap1 is a 15-kDa IgE-binding protein that is an isoallergenic protein variation (allergen nomenclature) [64]. PAN-1 is a protein that has two very distinct cytoplasmic and transmembrane domains. PAN-1 controls cell division and growth in plants by interacting with Rho GTPases. It is an intracellular process [65,66]. PAN-1 is essential for coat interactions during endocytic pathway changes in yeasts like *Saccharomyces cerevisiae*. Actin misregulation brought on by PAN-1 depletion results in actin flares that are associated with the cell’s coat instead of the dendritic membrane of sensory neurons [67]. In *C. elegans*, PAN-1 locates on the cell membrane and binds to the myelin regulatory factor (MYRF), a membrane-bound transcription factor that is essential for promoting synaptic rewiring [68]. Early larval ecdysis, the completion of the molting cycle during the adult molt in worms, and developmental activities that take place throughout the mid to late larval stages all depend on this protein [69]. It is an essential control point for the development of larvae and the progression of tissues during the transition from the larval to adult stage. It is unrelated to smell. We smoothly move to intracellular functions of PAN-1 with respect to actin, cell walls, membranes, and neural plasticity (see [14]). This is counter to the OR theory.

Given that 4CSPs have more than 100–200 amino acids and PAN-1 has more than 1000 amino acids, any conservation of function or shared ancestry should be highly questioned. However, PAN-1’s sequence match with 4CSP sequences is quite clear, since the N-terminus of PAN-1 clearly displays “4CSP” (Figure S3). When the 4CSP family is linked to protein families such as Thap1 and PAN-1, a chemosensory involvement, if any, may have emerged as a non-primary derived function.

Thap1s and Bommo4CSPs formed orthology groups. In particular, Thap1s built orthology groups with Bommo-4CSP14, Bommo-4CSP11, Bommo-4CSP5, Bommo-4CSP16, and Bommo-4CSP18. The phylogenetic tree that shows the relatedness between Bommo-4CSP and Thap1 has very strong bootstrap support values (98–100%; Figure S2B). Typically, Bommo-4CSPs and Thap1 have a 28–29% sequence identity. The 4CSP and Thap1 proteins are comparable at high-consensus and low-consensus residues (see sequence alignment; Figure S3). Similarly, the sequences of Bommo-4CSPs and PAN-1 show about ~37% identity. All critical residues and the four cysteines that make up the 4CSP family are among the high-consensus and low-consensus residues that PAN-1 and Thap1 proteins share (see sequence alignment; Figure S3). The possibility for non-primary functions to evolve is shown by the relatedness between 4CSP, Thap1, and PAN-1, especially considering that the proteins from these families may range significantly in size but share primary sequence identity (~28–37%). It is doubtful that non-primary functions have shifted to chemosensing for the 4CSP family, though. Bommo-4CSP10, a unique 4CSP with a 99% bootstrap value, a protein weight of 24.65 kDa, and a 211-residue sequence that terminates numerous prolines, is closely related to PAN-1 (see Figures S2B and S3). Given that proteins Bommo-4CSP10, PAN-1, and Thap1 are unrelated to chemosensing, it is quite likely that the proteins linked to them are, likewise, unrelated to chemosensing (see Table S1 and Figures S2 and S3).

### 4.4. 4CSP Evolution for the Appearance of Novel Phenotypes

In the context of 4CSP mutations for the emergence of a new phenotype, it is important to note that the polypeptide length of the protein between Cys29-Cys37 and Cys56-Cys59 ranges from 6–8 to 18–19 amino acids, depending on the insect species (Figure 1). For Culpi and Nasvi, the region between Cys29 and Cys37 contains a two-amino-acid residue insertion (Figure 1). Since Locmi also exhibits the same sequence variation, it cannot be linked to the formation of a specific phenotype [19]. However, an amino acid insertion (one residue) occurs in the region between Cys56 and Cys59 in Acypi, Apime, and Nasvi, which correspond to phenotypes that are drastically different from one another; the location of the insertion varies depending on the species (Figure 1A). Therefore, there are several insertions in Cys29-Cys37 that were probably present in the distant common ancestor of insects and had little to do with the emergence of a new phenotype, whereas other insertions in Cys56-Cys59 appear to be more connected to the development of a new phenotypic trait. Certain point mutations, in particular, seem to be very specific to a particular genotype. The jewel wasp genotype, Nasvi, for example, has several amino acid mutations (induced by insertion or deletion) in the 4CSP structure (Figure 1A). The degree of alteration in the 4CSP molecule is highly dependent on the insect species, as seen by the fact that Droso, Anoga, Aedae, Bommo, Trica, and Pedhu lack the insertions present in the other species (Figure 1A).

Given the number of mutations found in Bommo [26,27], it would be very interesting to look into the molecular impacts of the precise variation in gaps, insertions, and/or deletions throughout the species and between the tissues. Site-directed mutagenesis experiments that add or remove a specific amino acid residue or an entire amino acid motif should be carried out to check for loss of binding or protein function, especially if these mutations significantly change the protein’s intercysteine distance. To keep the protein structure stable, all 4CSPs have four cysteine (4-C) and two disulfide bridges (S1 and S2; Figure 1B). The length and makeup of the gap between the cysteine residues, however, vary substantially. Most changes occur in the spaces between α-helical domains (Figure 1B). None of the insect species from Droso to Pedhu had any notable changes in the vicinity of Cys3-Cys4 (S2; see Figure 1B). On the other hand, there is a high degree of mutations at S1, especially close to Cys2 (Figure 1B). This clearly shows the evolution of non-primary functions in this protein gene family and may provide a new role. Mutations in the intercysteine gaps of 4CSPs may have produced a unique insect phenotype—defined here as a novel behavioral, morphological, and/or physiological trait (Figure 1A,B).

## 5. The Evolutionary Trajectory of 4CSPs

The diversity, evolution, abundance, and number of *4CSP* genes are somewhat at odds with the new role in chemical communication. Numerous insect species have extremely few “4CSP”-coding genes, as evidenced by the four to eight *4CSPs* identified indifferently in Anoga, Apime, Droso, Nasvi, and Phedu [18,53,60]. The quantity of *4CSPs* is counted per two-four; odorant receptors (*ORs*) per hundred [70]. Does this mean that insects like bees, flies, lice, and wasps do not communicate chemically or that they have a bad sense of smell? In insects, there is only one ORCO receptor gene, and its function as a co-receptor and chaperone molecule in chemical communication is well established [71]. The term “odorant receptor co-receptor”, or “ORCO”, refers to the obligate OR coreceptor. As one of the most conserved ORs, ORCO produces a ligand-gated ion-channel complex that facilitates odor sensing by interacting with traditional odor ligand-binding receptors [71]. However, the situation with 4CSPs is different because it is believed that they transport odorants to ORs. If there are only four to eight 4CSPs as “chemosensory” proteins, this needs to be carefully considered.

Let us assume that the evolutionary path of *4CSP* genes—specifically, the path to a chemosensory role—can be explained by the small number of *4CSPs* seen in *Apis*, *Drosophila*, and *Pediculus*. Assume that even though honeybees are the most evolved pollinators and emerged last, along with the plants most suited to that role (~124 Mya [72]), six 4CSPs do not stop them from participating in it. When the pattern of six genes was preserved ~5.6 Mya for the divide between *Pediculus* lice [73], let us even assume that 4CSPs continued to be olfactory. *D. ananassae* has only three *4CSP* genes [18]. Even if fruit flies diverged between 5 and 62.9 Mya, at the time *ORs* diverged in primitive flies, before the diversification of the *Drosophila* and *Sophophora* subgenera (~40 Mya [74,75]), let us assume that even three 4CSPs would not stop them from engaging in olfaction. If this protein family is involved in chemical communication, these 4CSPs can only function as unspecific filters in this situation, assisting in the transportation of volatiles to ORs, as a number of species, such as Apime, Drome, and Pedhu, have extremely few *4CSP* genes. In this manner, a significant diversity of 4CSPs is not required because the selection of odorants is entirely non-specific. RNA editing and ribosomal peptide modifications, as detailed in Bommo, can result in a great diversity of 4CSPs [26,27]. This line of reasoning presents a very important question about the binding specificity of 4CSPs. Do 4CSPs belong to a class of proteins with low binding specificity? If so, how do other insects evolve with a lot more *4CSPs* (~20 in moths and beetles)? What led to the ~40–60 *4CSPs* that mosquitoes like Aedae and Culpi acquired? How does this address the olfactory problem for 4CSPs, given that the majority of these genes are exact duplicates of the same gene (see [18])? *D. ananassae* provides a model with only three *4CSPs* that allows us to quantify the extent to which the lack of a *4CSP* gene reduced the fly’s chemosensory capacity [18]. Fruit flies and many other hexapods are known to use a complex mixture of long-chain epicuticular hydrocarbons for chemical communication, pheromones, and mate recognition [76]. Drome communicates chemically through long-chain hydrocarbons (LCHs), but there are other cues as well. Short-chain hydrocarbons (SCHs) and other volatile chemicals or molecules (such as cis-11-vaccenyl acetate and cis-4-undecenal) can also function as chemosensory cues through gustation or smell [76,77,78,79]. How can all of these LCHs, SCHs, acetates, and aldehydes be handled by just three *4CSPs*? Most likely, a different class of genes that are more prevalent and much more closely related to olfactory cells is given the task.

For mediation of chemical communication in flies, the Che family, also known as “Chemosensory Proteins”, would be a better choice than 4CSP. First off, the Drome genome contains at least twenty *Che* genes and only four *4CSPs*. The Pfam-domain family of 4CSP (100–110 amino acids, 10–12 kDa, and 4 cysteines) is significantly different from that of CheAs and CheBs (>200 amino acids, >22.4 kDa, and >4–5 cysteines), suggesting different functions. While not all *Che* genes are engaged in pheromone detection, some *Che* genes, such *CheB42* and other *CheBs*, are sufficiently prevalent to be specifically involved in gustatory perception of pheromonal odors [80,81]. The *CheA75a* gene, on the other hand, is highly transcribed in Drome legs and wings and prominently expressed in the tarsal sensilla. CheA75’s ability to identify fungal spore-surface proteins suggests that it plays a role in immunological defense rather than odor pheromone recognition [82,83]. Therefore, whether a family of binding proteins is only involved in smell or is also involved in other physiological functions, including development and insect defense, seems to be a never-ending topic of discussion.

In particular, Che and 4CSP are at the center of this chemosensory function controversy. There is no shared evolutionary ancestry between 4CSPs and Che proteins. But both protein families—4CSPs and Ches—are rather unlikely to conduct chemical communication due to the small number of genes that encode them. Bees have neither *CheA* nor *CheB* and only six *4CSPs*. How do honeybees have an olfactory system designed to detect a wide variety of flower scents, semio-chemicals, LCHs, CHCs, and complex odorant mixes while they lack these two essential “chemosensory” protein gene families? In order to convey odors to ORs, 4CSP in Apime needs to be much more flexible than in Drome, since it needs to compensate for the absence of CheA and CheB. This means that it needs to bind a much wider range of odors. The ligand affinity profiles of 4CSPs from Apime and Drome should be compared with those of Acypi, Bemta, Bommo, Trica, Aedae, and Culpi. However, since 4CSP is widely found in the bee brain [53,84], we can simply argue that the ligand itself is not always odorous and that the structure of 4CSP is flexible enough to retain several molecules of the same ligand (see [7,8,9,10,11,12]). In the same way as taste receptors comprise GRs (gustatory receptors) and “some” IRs and olfactory receptors include ORs and “some” ionotropic receptors (IRs) [71], it is implausible that “some” 4CSPs—of which there are only six—evolved to perform this olfactory function in honeybees [53,70]. For this, “some” OBPs that are far more numerous than 4CSPs and/or other gene families—possibly simply the ORs—might be more helpful [85]. While honeybees’ olfactory systems are significantly expanded, their 4CSPs and Ches are not.

Confirmation of the involvement of a ligand-binding role in particular chemosensory processes, ORs/IRs (olfaction), GRs/IRs (gustation), and each 4CSP within a given species, like Drome or Apime, would require a great deal of molecular and cellular studies. One Apime 4CSP has been reported to bind the scent of bee-queen pheromone, but no coexpression with OR has been found, and this does not align with the protein’s widespread expression [86,87,88,89]. At this time, it is not possible to compare the binding affinities of every bee 4CSP with every 4CSP from Drome or Acipy. However, it is possible to draw comparisons between the gene structures and the *4CSPs*’ organization in the insect genome. This may make it easier to comprehend how *4CSPs* have influenced the evolution of insect phenotypes.

**Figure 2:**
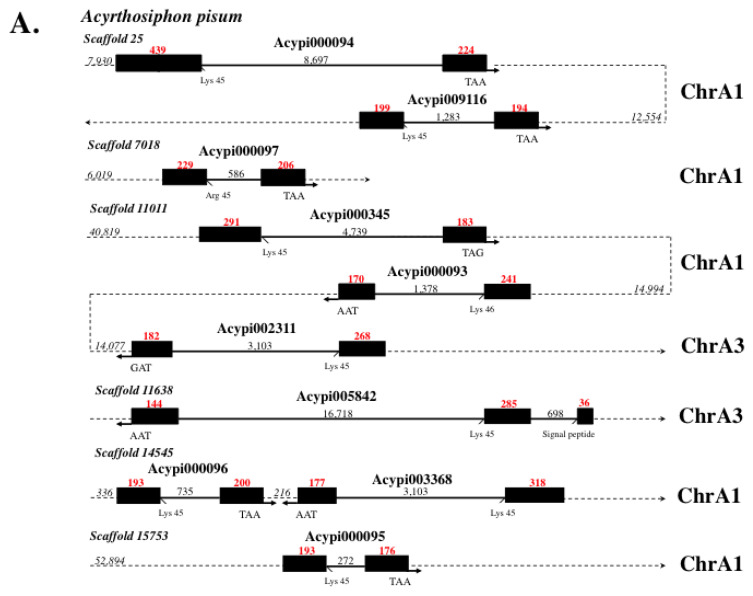

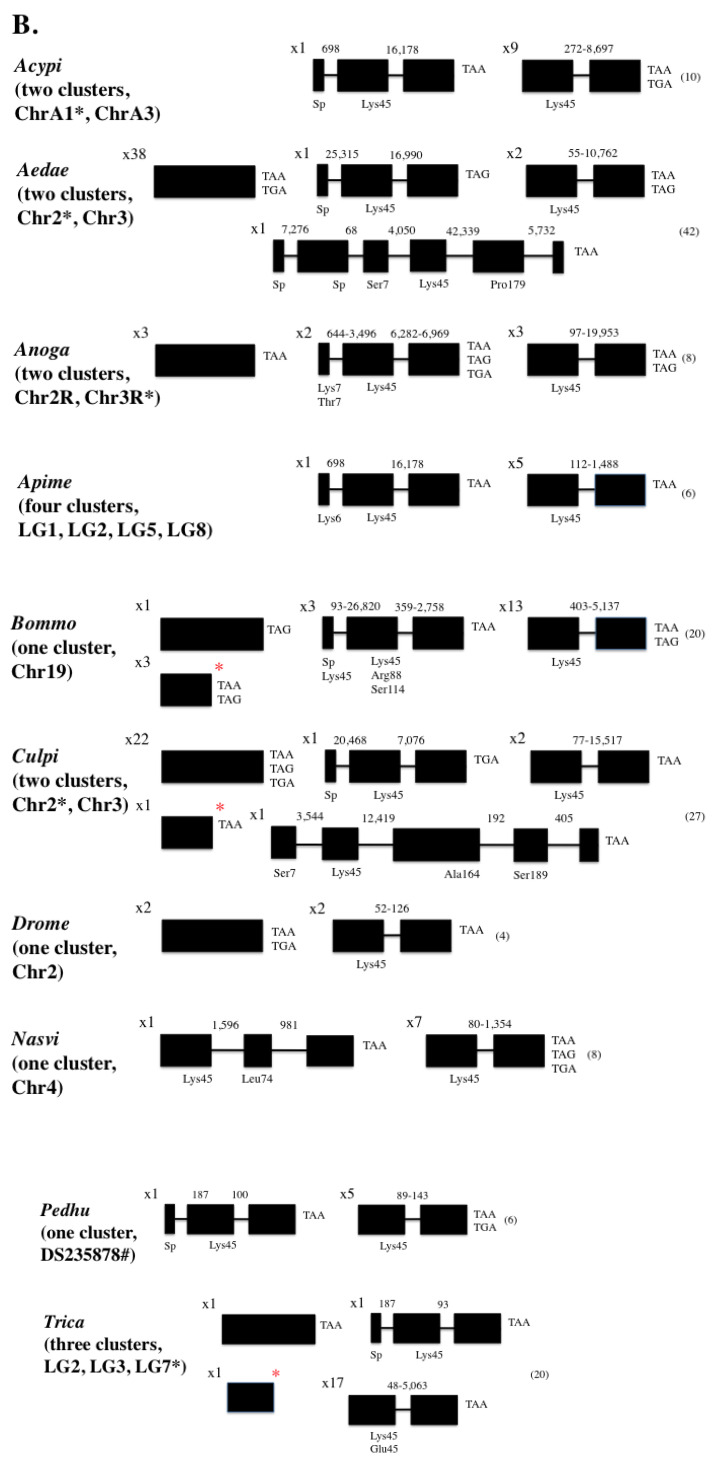
Ancient origin and common illustration of *4CSP* gene structures in insects. (**A**) Organization of aphid *4CSPs* on different scaffolds (25, 7018, 11,011, 11,638, 14,545, and 15,753) and the genomic *CSP* gene repertoire in Acypi (Table S1). Exons are shown as black boxes and introns as plain lines. The numbers above indicate the size of each segment of a gene, in base pairs, or intergenic distances (italics). The exon sizes are denoted by the numbers in red. The spacing between genes is indicated by dotted lines. The direction of the arrows—either 5′-3′ (right) or 3′-5′ (left)—denotes the orientation of the gene. Intron insertion sites are the residues at position 46 (after conserved Lysine45 or Arginine45). *Acypi-4CSP* genes have TAA or TAG stop codons, which are indicated by the stop codons in *Acypi000094*, *Acypi009116*, *Acypi000097*, *Acypi000093*, *Acypi005842*, *Acypi000096*, *Acypi003368*, and *Acypi0000095* (TAA), as well as *Acypi000345* and *Acypi002311* (TAG). The Acypi genomic DNA annotation provides the gene name, along with the gene’s location on the genome (genes are plotted onto scaffolds and genome assemblies without regard to function). (**B**) Comparative analysis of the arrangement of the *4CSP* genes in Acypi with Aedae, Anoga, Apime, Bommo, Culpi, Drome, Nasvi, Pedhu, and Trica. Comparison in the context of gene count, gene cluster arrangement, chromosomal dispersion, intron length, intron boundary, gene structure, and stop codon. Exons are shown as black boxes and introns as plain lines. Intronless genes are depicted as large black boxes. The intron size is indicated by the numbers above it, ranging from the shortest to the longest intron found in the species. In this species, the total number of *4CSP* genes found is indicated by the number in brackets. x1 indicates that the species possesses a single gene of this structure. x2 indicates that two genes of this structure have been found in the species, etc. The residues after conserved Lysine45 or Glutamic Acid45, as well as following Lysine7, Threonine7, Serine7, Leucine74, Arginine88, Serine114, Alanine164, Proline179, and Serine189, are found as intron insertion sites. Sp shows the intron in the genomic sequence coding for the signal peptide. Stop codons include TAA (Apime and Trica); TAA or TAG (Bommo); TAA or TGA (Acypi, Drome, Nasvi, and Pedhu); and TAA, TAG, or TGA (Aedae, Anoga, and Culpi). Under the species name, the number of clusters and the chromosomal distribution are shown in brackets. The black asterisk points to the main cluster. The asterisk in red (*) indicates pseudogene. # Chromosomes (Chr) have not yet been assembled from the Pedhu genome. Chromosomes assembled from updated genomes (July 2025).

Liu G. et al. (2020) reported that *4CSPs* are clearly grouped in pairs of duplicates on individual honeybee chromosomes [53]. A similar distribution of duplication on certain scaffolds is observed when analyzing *Acypi-4CSPs* (*Acypi000094*-*Acypi009116*, *Acypi000093*-*Acypi002311*, and *Acypi000096*-*Acypi003368*) (Figure 2A). Only 216 bps can separate the *4CSP* genes on the same scaffold, indicating co-expression, co-regulation, and synchronization (Figure 2A). Therefore, they may co-express and cooperate to regulate the same cell activity. Interestingly, the genomic DNA sequence of these *Acypi-4CSPs* is inverted (see Figure 2A). As was found for *Nasonia* [53], *Acypi-4CSP* duplicates typically point in different directions. As a result, their translocation rates are expected to be higher, as found in most species [90]. Inverted duplications (or gene inversions), the most prevalent kind of chromosomal rearrangement, have a significant influence on all facets of eco-evolutionary processes, not just sulfur odor chemosensing. These include mating systems, plant–insect interactions, the shift to herbivory, growth and survival on particular host plants, social organization, environmental adaptation, adaptation to temperature or climate change, reproductive isolation, and speciation. Inverted duplications like the ones we describe in the evolution of *Acypi-4CSP* genes are frequently linked to the appearance of a particular insect phenotype (see Figure 2 [91,92,93,94]).

It is evident from insect phenotypes produced by RNA interference that 4CSP is not involved in chemosensing but, rather, in the development of insect brain integument (see [84]). This makes a link between segmental duplications and 4CSPs’ function in neuronal connections. Neuron deletions, gene inversions, and genetically regulated duplications are crucial for the evolution of the brain. Gene inversion has played a role in the evolution of the primate brain [95]. Findings in spiders also show how gene inversions and duplications can drive neuronal innovation and the diversification of complex behaviors [96]. Therefore, considering gene inversions in *4CSPs* in relation to insect brain development is not aberrant. Based on the genetic information gathered from the bee brain, we hypothesize that 4CSPs have a broad role—not in odor transport but in environmental and host-plant adaptation, specific brain development, and/or new assemblage within the CNS (central nervous system)—as well as potential roles in the formation of new relays and neuronal connections with the antennae and the entire peripheral odorant receptor recognition system [53,84,97,98,99,100]. The degree of inversion duplications seen in the *Acypi-4CSPs* (Figure 2), as well as in the *4CSPs* from Nasonia, Bommo, Pedhu, Drome, and numerous other insect species [18,22,53,60,101,102,103,104], supports this, in addition to chromosomal inversions.

Like the majority of other insect genotypes, the *Acypi*-*4CSPs* are composed of two exons separated by a single, variable-length intron (Figure 2B). Similar to certain other *4CSP* genes from Aedae, Anoga, Apime, Bommo, Culpi, Pedhu, and Trica, *Acypi005842* contains an extra intron added a few nucleotides after the start codon that codes for amino acid methionine-1 (Figure 2A,B). This intron (phase 0 intron) is inserted after the third base; it does not interfere with the codon but changes the length of the signal peptide. This braces the notion put forth by Blobel (2000) that the functional importance of a molecule’s signal peptide is directly related to its length [105] and shows how strictly the signal peptide region’s splicing is controlled in *4CSPs* (Figure 2). The location of the 0 intron in the signal peptide may provide some insight into the roles of two distinct domains. The first six amino acids of 4CSPs may pierce the cellular or subcellular membrane. The additional amino acid residues of the signal peptide may be necessary for some molecular protein partners from the Signal Recognition Particle (SRP, protein–RNA complex) to properly localize and/or transit the 4CSP among the different cell systems [106,107]. Controlling the 4CSP molecule’s SRP may help understand its nature, which might be either as an internal protein in a multimolecular complex or as a secreted protein (in hemolymph). Similar to OBPs, 4CSPs are widely expressed from the sensory fluid to the inside of cells, according to immunocytochemistry studies, indicating that some 4CSPs (and OBPs) are stored within the cytoplasm, where they retain intracellular roles [14,53,108,109].

Is it possible that the 4CSP’s placement is determined by the gene structure or by the mechanism of splicing—or that intron *4CSPs* encode proteins that function intracellularly, while intronless genes generate 4CSPs that are discharged in the hemolymph? What is the ancestral function? Aphids, ants, bees, beetles, butterflies, flies, lice, mosquitoes, moths, and wasps all share a very ancient common ancestor, as evidenced by the fact that the introns in over 100 *4CSPs* and all other single-intron *4CSP* genes vary in length but are always found within the same location or boundary (Lysine 45) (Figure 2). The position of the intron boundaries in the *4CSPs* from whiteflies emphasizes this [52,110]. However, evidence of a gene’s deep evolutionary conservation can also be found outside of intron boundaries. Preserving the primary amino acid sequence, high-consensus residues, and stop codons is significantly more important. Splice junctions are necessary for the right polypeptide to be produced, and this also depends on the protein’s function. The genomic sequences of Acypi, Aedae, Anoga, Apime, Bommo, Culpi, Pedhu, and Trica contain introns in their signal peptides (see Figure 2). Because they highlight the gene’s ancient function and suggest that it has a very distant high evolutionary history, these 0 introns are likely significant. The class of insects is believed to have originated on Earth 480 Mya during the Ordovician, when terrestrial plants first emerged [111]. The *4CSPs* of all Hexapoda and insect species, including those of the orders Coleoptera, Diptera, Hemiptera, Hymenoptera, Lepidoptera, and Psocodea Phthiraptera, would typically be consistent with a shared heritage based on the available genetic evidence (Figure 2).

**Figure 3:**
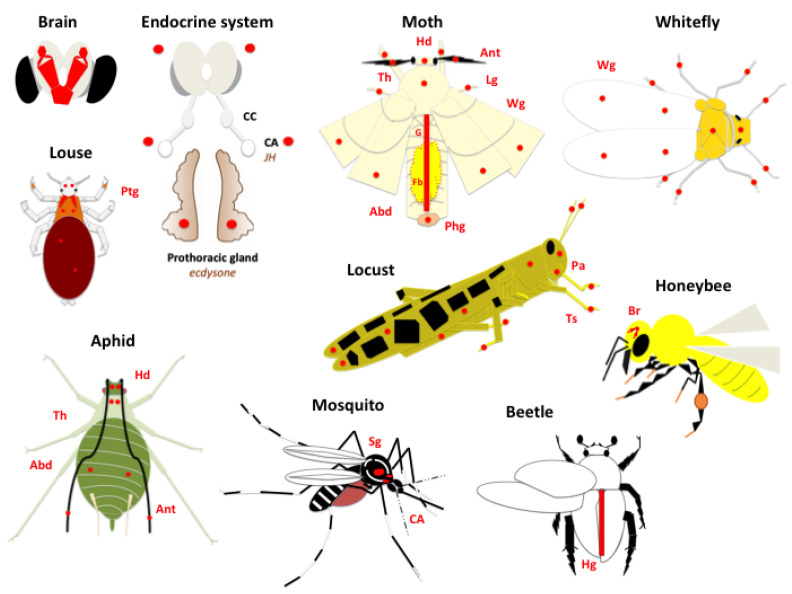
The general tissue expression profile of the protein family known as “chemosensory” (“4CSP”) in insects. The brain and endocrine system of insects express 4CSP. The location of 4CSPs is indicated by the red dots in aphid, beetle, honeybee, locust, mosquito, moth, whitefly, and locust. The primary structure (brain, gut, fat body, salivary gland, wing, and *Corpus allatum*) where 4CSPs are found in Coleoptera, Diptera, Hymenoptera, Heteroptera, Homoptera, and Phthiraptera is highlighted in red. Abd: abdomen; Ant: antennae; Br: brain; CA: *Corpora allata*; CC: *Corpora cardiaca*; Fb: fat body; G: gut; Hd: head; Hg: hindgut; Lg: legs; Pa: palpi; Phg: pheromone gland; Ptg: prothoracic gland; Sg: salivary gland; Ts: tarsi; Th: thorax; Wg: wings.

The history of the *4CSP* gene is old. It appears that *4CSPs* were present in the common ancestor of insects, which was probably the group of poisonous, blind crustaceans known as remipedes [112]. After that, each insect taxon at last began to show some distinctive patterns (see Figure 3). Unquestionably, distinctive behavioral patterns emerged as a result of distinctive phenotypic and functional traits (jointed legs attached to the thorax, aggregation, development of pairs of wings, acquisition of the ability to fly, antennal appendages, compound eyes, and sucking mouth parts). These traits evolved with flowering plants, especially in butterflies, wasps, bees, and many fly and beetle species (see Table S1 and Figure 1, Figure 2 and Figure 3 and Figures S1–S3).

## 6. Existence of 4CSPs Outside Insects and Hexapods

*4CSPs* might even be much older than remipedes, possibly dating back to the Silurian ostracods, which are minuscule (0.3 mm), bivalved, benthic crustaceans that are one of the oldest known arthropod groups (about >500 Mya) [113]. The evolution of insects, hexapods, and arthropods—likely from the early Myriapoda, which includes millipedes and centipedes (half a billion years ago)—as well as the evolution of cells in general, that is, cells from all species, including single organisms, bacteria, and yeasts, may have been impacted by mutation variations on *4CSPs*. It is obvious that bacterial homologous proteins should, likewise, be referred to as ‘4CSPs’ [61]. The development of a new nomenclature for this protein gene family, which must be used in all organisms, is crucial. The evolutionary analysis of *4CSP* genes in insects, crustaceans, and bacteria demonstrates unequivocally that this protein gene family predates the divergence of microbial cells (Bya) and did not originate from Insecta or Pancrustacea [61]. Horizontal gene transfer might have allowed Insecta and Pancrustacea to acquire *4CSP* from bacteria [61].

The incredibly broad tissue distribution of this protein family throughout the insect body is a powerful example of the pleiotropic significance of 4CSPs (Figure 3). Every single tissue in an insect’s body contains 4CSP. We conducted this experiment (see Figure S4), which was likely necessary around twenty years ago when *S. gregaria* (Schgr) gave the entire protein family the name and function of “chemosensory proteins”. In Schgr, “CSP” RNA signals from different tissues and developmental phases were amplified using real-time PCR (Figure S4), supporting the findings from Martín-Blázquez et al. [21]. It was found that none of the twenty “CSPs” in the silkworm are expressed exclusively in the antennae; instead, they are all widely distributed and should all be renamed [22]. This also holds true for whiteflies and bees [52,53]. Therefore, the tissue and development distribution of “CSP” is extremely broad across a large range of insect species. It can become quite challenging to identify a particular species or “animal” that expresses “CSPs” only in the antennae. The challenge may also be to find a “CSP” that is entirely exclusive to the antennae and directed to a particular OR or GR in order to maintain chemosensory specificity [114]. The idea of “chemosensing” would only be acceptable in this way. General concerns in our study include whether the proteins in the 4CSP family have many pleiotropic functions that are not limited to chemical sensing, whether their chemosensory function is not the primary one or applicable to only specific lineages, and whether 4CSPs are totally unrelated to chemosensing. The chemosensory function of these proteins is completely refuted when we look at their tissue distribution in both insects and crustaceans (see Figure 3, Figures S4 and S5).

Insects are not the only organisms that possess *4CSPs*. They are also expressed in a wide range of organisms, including many species of springtails, shrimps, crabs, lobsters, and copepods [61,115,116]. Given that 4CSP is found in every tissue of a crustacean’s body, the situation seems to be the same for all arthropods (Figure 3, Figures S4 and S5).

The expression of 4CSPs in the heart, hepatopancreas, brain, leg dactyls, eyestalks, gills, crawls, testis, stomach, ovaries, muscle, thorax, and Y-organ in crustaceans clearly suggests pleiotropy, as documented in insects [61]. The presence of many 4CSPs in copepod lipid sacs may indicate a function close to lipoid transport and storage, which may insist on pleiotropy (in which a gene is involved in a very general function that affects multiple phenotypic features), since lipids control a variety of physiological systems in insects and crustaceans [61,117]. Specifically, 4CSPs may have played a role in the change in tissue structure, as well as changes in cell-level development, size, form, and patterning. In order to form a new organ or even an entirely new organism, there may have been a combined morphogenesis using 4CSPs, involving the dynamic rearrangement of cells and neurons, as well as the complete reorganization of tissues (Figure 3, Figures S4 and S5).

It is interesting to note that the sea louse (*Calligus rogercresseyi*, Crustacea, Copepoda) has been the subject of preliminary and basic research on 4CSPs outside of insects [116]. A study reported binding to phenyl isothiocyanate and isophorone (a plant metabolite) over FA lipids [116]. It resembles the situation in *Bemisia* whiteflies, where Bemta-4CSP1 turns to lipids and Bemta-4CSP2 and Bemta-4CSP3 turn to small cyclic ketones [51,52,103]. 4CSPs may be able to bind lipids and nutritional metabolites in a variety of settings, including the early cells’ intracellular environment.

The existence of 4CSPs in bacteria strongly suggests this [60,61] (see NCBI locus CP023456, JALMGM010000101, JAAIFC010000213, JAAF01000214, JAAIFC0100000830, LYFD1000069, LYGG01000091, RBVL01000175, RBVL01000176, RBVL01000208, RBVL01000221, VUJW01000090, VUOA01000128, and WP_149730592 in multiple species). The ability of a bacterial cell to alter intracellular trafficking and molecular distribution is essential to its adaptability and resilience to environmental changes. Thus, the evidence against the chemosensory functions of 4CSPs is substantially strengthened. Comprehensive studies on functional assessments across taxa and experimental confirmation of proposed roles (lipoid transport, nutrition transport, toxin tolerance, and/or pathogenic activities) are required to properly establish the role of 4CSPs in bacteria. As functional investigations and discussions concerning the potential activities and evolutionary importance of bacterial 4CSPs have not yet been conducted, we will not go into more detail regarding the phylogeny of all bacterial 4CSPs here. Here, we show that *Bacillus* and *Klebsiella* sequences are identified in a phylogenetic group that contains 4CSPs from a wide range of insect species (Figures S2 and S3) and that insect 4CSPs and specific bacterial sequences can have up to 81–91% identity (average of 55% identity; Table S1). This finding highlights the 4CSP family’s non-chemosensory function and calls for more research into intracellular functions (see [14]). This claim is further supported by the fact that they are found in many different bacterial families [61].

Phylogenetic research, gene structure analysis, and the location of insect–bacteria clusters provide considerable support for the ‘qualitative’ claim that bacterial *4CSPs* are “evolutionary precursors” of insect 4CSPs [61]. The more ‘quantitative’ claim that bacterial 4CSPs have a conserved function is supported by sequence identities ranging from 55% to 91% (see Figures S2 and S3 and Table S1). Before attributing a 4CSP to an olfactory function, it is crucial to take into account the fact that many bacterial 4CSPs are “twins” of insect 4CSPs [60,61]. When looking for the function of these proteins, insect–bacteria comparison are crucial (up to 91% identity; see Table S1). Although this needs to be demonstrated experimentally, being “twins” suggests that, from a biological standpoint, their roles—such as adaptation—are most likely the same. Investigation of the functional evidence for bacterial 4CSPs is urgently needed. The insect–bacteria similarity in 4CSPs contradicts established chemosensory paradigms rather than supporting an olfactory role for 4CSP [61].

We conducted a BLASTP (version number 2.17.0) investigation in the Microbial Protein Database using Acypi-, Bommo-, and Pedhu-4CSP as a template. The *Sorangiinae* bacterium (WXB18440, Locus LZC94_14500; see Table S1), *Actinoallomurus acaciae* streptosporangiales (WP_378213916), and *Shewanella shenzhenensis* (MCH1932606) all have 4CSP sequences (46–59% similarity). These microbes are endophytic, myxobacterial, soil, and tree-root bacteria that either consume insoluble organic materials or are so prevalent in soil and water that they can be found on sea sediments. Plant odor components and airborne volatiles are absent from the marine environment. The chemical components that give the deep ocean its smell—dimethyl sulfide and bromophenol from algae—are entirely distinct from those that give off plant and insect odors. We suggest that 4CSPs, rather than olfaction, are present in bacteria, insects, and crustaceans because they aid in their adaptation to a wide range of environmental conditions.

## 7. Expression Profiling in Organisms, Development, and Tissues

In single-cell organisms, chemotaxis for glucose and phenol can be regarded as a component of gustation, olfaction, and chemical senses. The fact that so many bacteria express 4CSP homologs is therefore not surprising. However, considering the remarkable similarities between insect and bacterial 4CSPs and the fact that all Bommo-4CSPs are expressed outside of the sensory organ paradigm (mainly in the gut and fat body), it is rather inconsistent to refer to a whole family as “chemosensory proteins”.

It is already known that bacteria have chemical sensing proteins, which are entirely distinct from 4CSPs. Methyl-accepting chemotaxis proteins, or MCPs, are a family of proteins that identify chemical gradients in bacteria. These protein receptors can recognize a variety of chemicals [118]. Within a bacterial cell, thousands of MCPs and signaling proteins form a densely packed network through distinct protein–protein interactions. The amount of 4CSPs (about four) is far less than the thousands of MCP I and MCP II molecules per cell found in bacteria [119]. Bacterial cells also contain “Che” proteins. One of their roles has been the control of bacterial cell adhesion [120]. By interacting with periplasmic space proteins, MCPs can either directly or indirectly bind attractants or repellents to activate Che proteins. A number of protein reactions may be triggered by this [121]. This system is unrelated to the molecular underpinnings of insect olfaction. Compared to MCPs, which are aspartate receptors arranged as trimers of dimers [121], insect ORs [71] have a very different structure, and the environment–MCP receptor interface lacks any intermediate soluble proteins. But just like insects, bacteria use protein–protein interactions, supramolecular complexes, intracellular transmitters, phosphorylation, and lipid transport to transduce signals. Che proteins are activated in the cytosol after signals from MCP receptors cross the cell membrane [122,123]. Therefore, as part of the signaling cascade between the “odor” and the flagellar motor switch, cytoplasmic proteins such as Che proteins modify the tumbling frequency and the receptor responses. It is necessary to thoroughly explore the role of 4CSP in this intracellular cascade, keeping in mind that while Che protein is unambiguously assigned the chemotaxis function, 4CSP may have a completely distinct function from that of Che.

It would also be somewhat nonsensical to call these proteins “chemosensory”, that is, proteins that convey odors to olfactory neurons, given their widespread distribution across the tissues of insects and hexapods (see Figure 3 and Figure S5). Along with numerous other crab species, Achelata 4CSPs are found in phyllosoma, larval and adult stages, and a range of non-sensory organs in the adult stage (see Figure S5 [61]). In addition to the sensillum, 4CSPs are also present in insect venom and pheromone glands [26,124]. About sixteen 4CSPs are expressed by the sex pheromone gland of the silkworm that uses bombykol as its only sex pheromone component [26]. 4CSPs are present in salivary secretion and other fluids, in addition to wasp venom and the pheromone gland of female moths. The ovaries, wings, legs, mouth, mandibles, proboscis, cephalic capsula, eyes, head, thorax, abdomen, epidermis, fat body, and gut are among the tissues that express 4CSP. These tissues are mostly covering and metabolic tissues; they are not olfactory [22,52,125,126,127,128,129].

We then examined the expression of 4CSPs in various tissues across various arthropods using Expressed Sequence Tags (ESTs) for comparison with molecular investigations and to collect further tissue distribution data. By definition, the insect EST databases (BeeBase, BeetleBase, FlyBase, KAIKO/SilkDB, Hymenoptera Genome Database, and VectorBase) contain over 30,000 mRNA sequences from different tissue libraries and offer information on the connections between proteins and tissues of origin [130,131,132,133,134,135]. Determining where 4CSPs are expressed in the insect body can undoubtedly provide information on their function. If we found that they were limited to the antennae, chemosensory function would be supported. Similar to many other insect species, an examination of the EST database in Pedhu and Acypi reveals that 4CSPs are not limited to the antennae (Figure 3). Six transcripts from first-instar Pedhu larvae and engorged adults were used to identify the 4CSPs in the louse body (Table S1 [60,136,137,138]). According to EST transcript sequencing data from The International Aphid Genomics Consortium (2010) [139,140,141], Acypi-4CSPs are also found in several tissues, including the head, thorax, abdomen, and antennae of third-instar nymphs, as well as in winged and wingless parthenogenetic females that have either been treated with ampicillin or have been inoculated with bacteria to eliminate pathogens. *4CSP* expression in Acypi, Apime, Aedae, Anoga, Bommo, Culpi, Drome, Nasvi, Pedhu, and Trica was studied using specific insect databases, such as AphidBase, BeeBase, BeetleBase, Flybase, KAIKO/SilkDB, Hymenoptera Genome Database, and VectorBase [18,22,53,103,130,131,132,133,134,135,136,137,138,139,140,141]. Examining EST-cDNA libraries and databases from over ten different insect species confirms Northern blot, Western blot, PCR, and real-time PCR results from molecular studies that analyzed a variety of tissues in addition to antennae: 4CSP is extensively dispersed throughout the insect body, from the sex pheromone gland to the prothoracic gland and the brain (Figure 3).

In insects, the labellum, legs, wings, pharynx (pharyngeal taste organs), and external reproductive organs all include gustatory neurons. Chemosensory neurons and/or receptors are therefore likely present in many of the organs that express 4CSP shown in Figure 3. However, the brain, hindgut, fat body, CA, many glands, and muscles—the primary organs where 4CSPs are produced in insects—seem unlikely to include chemosensory neurons and/or odorant receptors. Shrimp and lobster interior (brain, suboesophageal ganglia, abdominal muscle, heart, hepatopancreas, and molting gland) and exterior (claws) segments contain 4CSPs (see Figure S5) [61]. Chemosensing has nothing to do with these tissues. Figure 3 and Figure S5 show the tissue distribution of the 4CSP family in both insects and hexapods; this is not the tissue expression profile one would expect from a family of “chemosensory” proteins.

## 8. To Design the “OS-D/CSP” Pfam Domain, New Terminology Is Needed: Structure or Function?

Typically, a neuropeptide or neurohormone molecule is named after the first “physiological” or “pharmacological” effect that is noted for it; however, this naming scheme often turns out to be pretty wrong. The best course of action is probably to rename all naturally occurring peptides, hormones, carrier proteins, transporters, binding proteins, and related molecules in a more objective, methodical, and unbiased way. Like JH or “PBAN/MRCH”, this binding protein family’s expression site presently defies the names “CSP”, “OS-D”, and “SAP” due to its diverse range of functions, which range from pleiotropy to various phenotypic expressions (see Figure 3 and Figures S1–S5). New names or nomenclature must be built, but they cannot be limited to a function, chemosensory, or olfactory structure; take on any enigmatic roles; merely rely on overly speculative conclusions; or continue to rely on the absence of enough evidence or conclusive proof. At the very least, the name should take into account insect physiology, organismal phylogeny, orthology, conservation of the functional domain, evolution of pleiotropy, the number of loci, gene expression, the developmental profile, localization, and tissue distribution (see Table 2). The term “chemosensory protein” is rejected by tissue distribution that deviates from the chemosensory paradigm (Figure 3). Because these proteins are not “exclusively expressed in olfactory cilia”, as the current terminology implies, the terms “OS-D”, “CSP”, and “SAP”, which stand for “Olfactory Specific-type D”, “Chemosensory Protein”, and “Sensory Appendage Protein”, respectively, become rather odd and inaccurate when used to refer to the protein family. For the sole expression in olfactory cilia, this is untrue. The designations of “OS-D”, “CSP”, and “SAP” are completely inappropriate when referring to this family of proteins expressed in non-sensory metabolic organs and tissues, as well as all those that appear to have spread from bacteria and viruses to insects and hexapods. Given the tissue distribution from eggs to the fat body, “JH-related proteins”, or “JHRPs”, would be a far better moniker for these proteins than CSPs, OS-Ds, OBPs, or SAPs. It is unquestionably wrong and incorrect to tune the NCBI Pfam-domain A10/OS-D directly to a “chemosensory” protein without considering the expression data.

There are several reasons why a contentious discussion is no longer necessary: (1) Hexapods, insects, and bacteria have all had their EST and genome databases analyzed. The EST-RNAs of bacteria, insects, and hexapods show that none of these “OS-D/CSP” proteins is specifically tuned to olfactory and/or taste chemosensory organs, which is consistent with the molecular expression data (Figure 3 [22]). (2) They all support RNA interference and gene knockdown studies. Eliminating the “*CSP*” has no effect on smell but has an impact on insecticide/pathogen response and/or how the insect brain develops [84,142]. (3) “CSPs” respond to chemical, bacterial, or viral infections. They react to more than only insecticide compounds. They participate in general immunity [22,42,43,52,53]. (4) “CSP” binds to linoleic acid (LA), which is essential for immunological response and development [52]. (5) The “OS-D/CSP” Pfam domain is found in general intracellular elements that are involved in nuclear gene regulation, transcription, actin, and cell walls rather than olfaction (see [14]).

The scenario appears to be comparable to that of a superfamily of widely distributed, heterogeneous proteins known as “lipocalins”, which are responsible for the transport of small hydrophobic molecules such as lipids and steroids (the term ‘lipocalin’ comes from the Greek lipos, which means fat, and the Greek kálux (κάλυξ), which means cup).

One notable distinction, though, is the diversity and poor conservation of lipocalins across the course of evolution [143]. Conversely, the “CSPs” are relatively homogeneous proteins with protein structures similar to those that are present in bacteria, insects, and hexapods [61]. CSPs are similar in terms of intron boundaries, evolutionary well-conserved molecular architecture (such as a lengthy hydrophobic tunnel in a prism with a pattern of four cysteine residues that are always in the same position), and tissue expression profiling (ubiquitous expression). But the primary distinction between lipocalins and “CSPs” may be that the latter are associated with intracellular elements, nuclear transcriptional factors, and cytoskeleton actin linkers that lipocalins do not yet cover (see [14]).

The major function of “Chemosensory Proteins” in insects—which is thought to be the extracellular stimulation of ORNs—remains rather ambiguous because there is no proof of a CSP–OR link. Using “CSPs” to build this protein family is additionally misleading because the same name is used to design two other distinct protein families. “CSP” also refers to the *Clostridium difficile* spore germination Csp family of proteases and bacterial “Cold Shock Proteins” [144,145]. Here, we initiate a new discussion on the possible functions of “CSP” in cell lipoid trafficking, taking into account various insect and hexapod tissues, intracellular localization, and evolution in viruses and microbes (Figure 3 [14,61]). We call for the use of a new nomenclature to designate this protein family. As proteins that contain lipoid ligands, similar to lipocalins, it would be reasonable to refer to “CSPs and OBPs” as “*lipoclistins*” (see Steinbrecht [146]). The primary structural element of the “CSP”, an open-air structure, would be best characterized as “*lip-anoiktins*” (open = ἀνοικτός/anoiktós) according to this naming scheme based on the Greek ancestry of terms and names. According to Steinbrecht [147], the naming strategy based on the “Latin” root of words and names implies that the Latin term “*apertus*” (open) would also be a suitable way to refer to the “CSP” protein family.

**Table 2:** Changing the name of “ChemoSensory Proteins” to reflect their molecular structure, tissue-distribution, and intracellular functions.

Name	Organism	Authors	Year	Reference
Lipoclistin	*Insects*	Steinbrecht	2003	[146]
LA-BP	Bemta	Liu et al.	2016	[52]
Phenyl-BP	Bemta	Liu et al.	2016	[52]
Lipid-BP	Bemta	Liu et al.	2020	[53]
JHRP	Aedae	Picimbon	2020	[18]
Mucin module	Aedae	Liu et al.	2026	[14]
TIF module	Aedae	Liu et al.	2026	[14]
ASRP module	Aedae	Liu et al.	2026	[14]
NPCP module	Aedae	Liu et al.	2026	[14]
4CSP *	*Insects, crustaceans, and microbes*	Liu et al.	2026	[This reference]

* Different meaning of the abbreviations, but the similarity makes it easier to adjust.

A generic name like “*arthrolipin*” would be inappropriate for a “CSP” molecule, since it is not specific to the arthropod family; it can also be found in viruses, bacteria, and a variety of prokaryotic cells [61]. Not to be forgotten, in honor of the early literature and the old name, Alejandro P. Rooney proposed “*4CSP*” (*Four Cys Soluble Proteins*) or *CSP/4Cys* without drawing any broad conclusion regarding their physiological function, which would be the most sensible name for the protein family.

There are very few examples of proteins other than 4CSPs that also include four cysteines, aside from the zinc-binding protein of the Ecl1-type and the DNA-binding protein of nuclear factor I [148,149]. Cytokines, on the other hand, are categorized into multiple sub-families of chemokines according to the locations of successively conserved Cys residues (Cys-Cys, X-Cys, Cys-X-Cys, and Cys-X-X-X-Cys) [150,151]. While Cys-Cys chemokines contain the cysteines exactly next to each other, Cys-X-X-X-Cys (or CX3C) chemokines have three amino acids between two cysteine residues [150,151]. The N-terminal region of chemokines has all conserved cysteines, which clearly sets them apart from 4CSPs, even though the two protein families retain the same number of Cys residues. Then, discussing Cys’s position is not all that interesting. Our data suggests that the four-cysteine profile may be crucial for intracellular function and/or contact with DNA [14], which may be even more interesting than finding 4CSP throughout the entire insect body.

It is very confusing to call a protein “chemosensory” when it is produced in CA and CC. Would it be more confusing if any members of the 4CSP family acquired six, eight, or ten cysteine residues—the kind seen, for example, in “nonsensory OBPs”? The A10/OS-D Pfam domain is what 4CSP refers to. Given that they include this Pfam domain, it is likely that big intracellular proteins like PAN-1, ASRP (actin skeleton regulatory protein), NPCP (nuclear pore complex protein), Mucin, and Transcription Initiation Factor (TIF) were constructed by adding additional cysteine to “CSP/OS-D”, see [14]. Renaming the A10/OS-D domain to the 4CSP module and eliminating the term “chemosensory” would be beneficial for all these distinct families of intracellular proteins, which have diverse names, acronyms, and functions but share one essential element—the 4CSP module at the N-terminus [14].

## 9. Concluding Remarks

Chemical sense-related lipophilic ligand-binding proteins (CRLBPs), putative pheromone-binding proteins, OBPs, and/or chemical binding proteins (CBPs) are names given to certain genes and proteins found in insect genomes (in *Drosophila*: ~51 OBPs, two subfamilies of CRLBPs, and ~20 CBPs [70,152,153,154,155]). The capability of CRLPs, PBPs, OBPs, and CBPs to deliver hydrophobic or hydrophilic chemical impulses to specific odor or taste receptors is a shared trait. They are therefore usually found in the olfactory and taste systems, including the pheromone-sensing gustatory hairs of insect tarsi and palpi, as well as the front legs, wings, and antennae [152,153,154,155]. Here, we are focusing on a carrier protein with a distinct functional structure—four cysteines, about 10–12 kDa—that relates to the NCBI’s A10/OS-D Pfam domain.

We have reassessed the function of “CSPs”, which is extremely diverse, with the goal of shifting the paradigm from a strictly “chemosensory” function of this binding protein family to a larger one that is not limited to a putative odor-binding function but, rather, has a variety of biological purposes in insects and hexapods. Currently, the nomenclature used to refer to these proteins as “chemosensory” proteins is utterly incorrect and disjointed. This is even worse now, given our current level of knowledge, compared to twenty years ago, when we were limited to thinking about expression in the antennae, ignoring the expression in the brain, CA, CC, thorax, and abdomen. This protein family, which is widely expressed in the insect gut, fat body, thoracic glands, and *corpora allata*, is also linked to immune responses and intracellular systems and cannot be categorized as “chemosensory”.

After almost two decades of study, there are two crucial aspects that should no longer be overlooked: (1) 4CSP has a pretty broad substrate selectivity, meaning that it can bind phenyls, transcription regulators, and/or a range of other intracellular components, in addition to lipoid molecules. Even the possibility that 4CSP binds to nucleotides, DNA, RNA, and/or other nuclear components can be studied ([14]). (2) It is not exclusive to sensory appendages. It can even be found in microorganisms (see [61]). Therefore, “OS-D”, “CSP”, and “SAP” are no longer appropriate names for this protein gene family. Since the evidence for a particular function can be quite weak and prone to excessive change and disagreement, even across related groups of proteins, the new name should ideally not allude to any particular functional role. We propose 4CSP as the first example, a protein family that has long been considered “chemosensory” and that would be better defined or classified based on its structure rather than a particular function. This is especially true when chemosensing, the sole function of the protein family, has been the focus of such a long-running debate and has been the topic of so much research without being conclusive.

Would it be possible to develop a software algorithm that would instantaneously identify a protein from Insecta or Hexapoda as belonging to this 4CSP family and/or a particular phenotype based on the presence of these distinctive four cysteine residues, without attributing it to a very elusive “chemosensory” function? Such a strategy (either in addition to or instead of BLAST (2.17.0) in NCBI) would make sense because there are hundreds of known 4CSP sequences and more to come among the more than ten million insect species on Earth. The NCBI database’s current algorithm classifies everything in the Pfam A10/OS-D domain as “chemosensory protein”, which is totally incoherent, since it does not refer to the analysis of 4CSP’s tissue distribution, ontogeny, phylogeny, evolutionary relatedness, and potential intracellular functions. We should build a new algorithm that prioritizes structure and, eventually, RNA mutations in order to classify new clones instead of depending on a very elusive function. Although handling insect bioinformatics would be challenging, it would be quite beneficial if we considered the fact that OBPs, for instance, have a somewhat distinct cysteine profile (six cysteine residues). Therefore, we could immediately connect a new clone with one of these protein families by developing two algorithms that are specifically tailored to the 4-Cys or 6-Cys pattern. There would be a substantial and powerful advantage to the ability to provide more accurate details regarding protein identification, such as the size and composition of intercysteine gaps, the quantity (and location) of extra-Cys residues, and the Cys pattern. This would prevent spin from spreading throughout public databases. Point mutations such as +Cys and +Gly would be immediately highlighted. The software would arrange the protein sequences based on gaps, conserved features, deletions, inserted amino acids, or specific protein motifs using this mutation data. Any program that could construct a tree and provide information on rooting, bootstrapping, the number of cysteines, the length of interCys gaps, and the marking of particular point mutations on the branch would be far more sophisticated than BLAST, BEAST, IQ-tree, MOLPHY, PAUP, and other phylogenetic software. This would benefit molecular biology, protein biochemistry, and gene evolution far more than putting a clone detected in *corpora cardiaca* cells under a “chemosensory” classification.

In the future, it would be more rational and interesting to compare Gram+ and Gram- bacteria in order to investigate the ability of 4CSPs to bind ligand molecules such as adenosyl methionine or homoserine lactone rather than smells. It would also be interesting to investigate the binding characteristics and diversity of 4CSPs in axenic insects, since these insects do not have bacterial communities in their digestive tracts. Fecal bacterial 4CSPs in axenic or gnotobiotic insects, such as cockroaches and caterpillars, are unlikely to transmit pheromone signals or odorants. Importantly, many bacteria produce 4CSPs that resemble those of insects, and the insects’ 4CSPs are usually located throughout the entire body rather than just on the antennae. Finally, there is no evidence that any of the Bommo-4CSPs bind to volatile carboxylic acids (VACs), volatile organic compounds (VOCs), worm cohesion pheromones, and/or specific bacterial odors. Exposure to pesticides resulted in a significant and noticeable upregulation of 4CSPs in the Bommo-Avermectin experiment, which examined several dissected tissues instead of just the antennae [22]. This type of result promotes a new “metabolic paradigm” in microbe and insect research in place of the “chemosensory paradigm” when discussing the general function of 4CSPs.

## Figures and Tables

**Table 1:** Historical naming of the family of “Four-Cysteine Soluble Proteins” (“4CSPs”).

Name	Organism	Authors	Year	Reference
p10	Peram	Nomura et al.	1992	[54]
A10	Drome	Pikielny et al.	1994	[55]
OS-D	Drome	McKenna et al.	1994	[56]
Ebsp-3/PebIII	Drome	Dyanov et al.	1995	[57]
Pam	Peram	Picimbon & Leal	1999	[3]
CSP	Schgr	Angeli et al.	1999	[4]
SAP	Manse	Robertson et al.	1999	[58]
Pherokine	Drome	Sabatier et al.	2003	[42]
Mp10	Myzpe	Bos et al.	2010	[59]

## Data Availability

The original contributions presented in the study are all included in the article/Supplementary Materials. Further inquiries can be directed to the corresponding author.

## Supplementary Materials

The following supporting information can be downloaded at: https://www.mdpi.com/article/10.3390/insects17020202/s1, Figure S1: PAUP*10Altivec phylogenetic analysis of Acypi, Bommo, and Pedhu 4CSPs; Figure S2: PAUP*10Altivec phylogenetic analysis of Acypi, Bommo, and Pedhu 4CSPs, along with PAN-1 and Thap1 proteins; Figure S3: Alignment of PAN-1 and Thap1 amino acid sequences with Acypi, Bommo, and Pedhu 4CSPs; Figure S4: Tissue distribution of *S. gregaria* 4CSP1. Figure S5: 4CSP distribution profile in crustaceans’ tissues; Table S1: Aphid ‘4CSP’ gene repertoire in comparison to that of moths and lice, genome assembly, and protein identity.

## Abbreviations

A10	Antennal protein A10
4-C	4-Cysteine
4CSP	4-Cysteine Soluble Protein (“CSP/OS-D”)
6CSP	6-Cysteine Soluble Protein (“OBP”)
Acypi	*Acyrthosiphum pisum* (pea aphid)
Aedae	*Aedes aegypti* (dengue yellow fever mosquito)
Anoga	*Anopheles gambiae* (malaria mosquito)
Allergen Tha p 1	IgE-binding protein (15 kDa) and major allergen of pine processionary caterpillar (*Thaumetopoea pityocampa*, Thapi)—variant 1.0101
Apime	*Apis mellifera* (honeybee)
ASRP	Actin skeleton regulatory protein
BEAST	Bayesian evolutionary analysis sampling trees
BLASTp	Protein BLAST
Bommo	*Bombyx mori* (silkworm moth)
Bemta-CSP1	*Bemisia tabaci* “Chemosensory Protein”-1 (LA-binding protein)
CA	*Corpora allata*
CBP	Chemical Binding Proteins
CC	*Corpora cardiaca*
CHC	Cuticular hydrocarbons
Che	Chemotaxis proteins (histidine kinases)
CheA, CheB	Chemosensory proteins-A, Chemosensory proteins-B (*Drosophila*)
Chisu	*Chilo suppressalis*
CNS	Central nervous system
CRLPB	Chemical sense-related lipophilic ligand binding proteins (*Drosophila*)
CSP	ChemoSensory protein
CSP	*Clostridium difficile* spore germination protease
CSP	Cold Shock Protein
Culpi	*Culex pipiens* (common house mosquito)
CX3C	3 amino acids (X) between two cysteine residues
Cys	Cysteine
Drome	*Drosophila melanogaster* (fruit fly)
Droso	*Drosophila* species
EA	Elaidic acid
Ebsp	Ejaculatory bulb-specific protein
EST	Expressed Sequence Tag
FA	Fatty acid
GR	Gustatory receptor
GTP	Guanosine triphosphate
Holob	*Holotrichia oblita*
IgE	immunoglobulin E
IQ-Tree	Phylogenetic inference using maximum likelihood software package
JH	Juvenile hormone
JHRP	Juvenile hormone-related protein
KAIKO	Silkworm genome database (2009)
Ki	Inhibitory constant
LA	Linoleic acid (C18:2)
LCH	Long chain hydrocarbon
Locmi	*Locusta migratoria* (migratory locust)
LRR	Leucine-rich repeat protein complex
Manse	*Manduca sexta* (tobacco hornworm, sphinx moth)
MCP	Methyl-accepting chemotaxis protein
MOLPHY	Molecular Phylogenetics program package
MP	Maximum parsimony
Mp10	Myzpe p10-like protein
MRCH	Melanization and Reddish Coloration Hormone
MYRF	Myelin regulatory factor
Myzpe	*Myzus persicae* (green peach aphid)
Nasvi	*Nasonia vitripennis* (parasitoid jewel wasp)
NMR	Nuclear magnetic resonance
NPCP	Nuclear pore complex protein
NPN	1-N-phenylnaphthylamine
OBP	Odorant-binding protein
OR	Olfactory receptor
ORCO	Conserved Odorant Receptor type (Co-receptor)
OS-D	Olfactory Specific-type D protein
p10	Peram 10 kDa protein from regenerating legs
PAN	Hexameric ATPase complex
PAN-1	Protein encoded by the pan-1 gene in the nematode *Caenorhabditis elegans*
PAUP	Phylogenetic analysis using parsimony
PBAN	Pheromone Biosynthetic Activating Neuropeptide
PCR	Polymerase chain reaction
Pedhu	*Pediculus humanus humanus* (human body louse)
Peram	*Periplaneta americana* (American cockroach)
PBP	Pheromone-binding protein
PebIII	Protein from ejaculatory bulb-type 3
Phk	Pherokine (hemolymph protein)
Ras	Family of GTPases derived from rat sarcoma virus
Rho	Family of GTPases, family of small (~21 kDa) signaling G proteins, subfamily of the Ras superfamily
RhoGAP	Rho GTPase-activating protein, regulator of the Rho-related protein family, crucial in many cellular processes, motility, contractility, growth, differentiation, and development
Rippe	*Riptortis pedestrus*
RNAi	RNA interference
Rt-PCR	Real-time polymerase chain reaction
SAP	Sensory appendage protein
SCH	Short chain hydrocarbon
Schgr	*Schistocerca gregaria* (desert locust)
SDS	Sodium dodecyl-sulfate
SilkDB	Silkworm Knowledgebase
SRP	Signal recognition particle
TIF	Transcription initiation factor
Trica	*Tribolium castaneum* (red flour beetle)
UPGMA	Unweighted pair group method with arithmetic mean
VAC	Volatile carboxylic acid
VOC	Volatile organic compound
Y-organ	Paired glands that secrete ecdysteroids in crustaceans
