# Supplementary material for: Renaming the ‘OS-D/CSP’ Family (Part 1): ‘4-Cysteine Soluble Proteins’ (4CSPs)—Molecular Nomenclature, Structure, Expression, Evolution, Tissue-Distribution, and Pleiotropy
Source: Insects. 2026 Feb 13;17(2):202. doi: 10.3390/insects17020202 (PMC12940638; doi:10.3390/insects17020202)
Supplement: Supplementary file 1 [file insects-17-00202-s001.zip › Liuetal.Insects2025-Part1-FigureS3.pdf]

```
                  1        10        20        30        40        50        60        70        80        90        100       110       120       130
                  |--------+---------+---------+---------+---------+---------+---------+---------+---------+---------+---------+---------+---------|
             Mp10 GPAYTTKYDHIDIDQVLGSKRLVNSYVQCLLDKKPCTPE-GAELRKILPDALKTQCVKCNATQKNAALKVVDRLQRDYDKEWKQLLDKWDPKREYFQKFQQFLAEEKKKGVVKF
      Acypi000097 GTAYTTKYDHIDIDQVLASKRLVNSYVQCLLDKKPCTPE-GAELRKILPDALKTQCAKCNATQKNAALKVVDRLQRDYDKEWKQLLDKWDPKREQFQKFQQFLAEEKKKGVVKF
       BonmoCSP10 MPKYDERYDYLDVDDIFRNKRLVRNYVDCLINAQRCTPE-GKALKRILPEALRTKCIRCTERQKRTSVKVIRRLKNEYPEEWAKLASRWDPTGDFTRYFEDYLAKEHFNTIPGSGPTVNVLSLQTTPPPP
        HelzePAN1 MPKYDSRYDYLDVDAVFTNKRLVRNYVDCLINAVRCTPE-GKALKRILPEALRTKCVRCTERQKRTAVKVIKRLKNEYPDEWAKLASRWDPTGDFTRYFEEFLAKEQFNTIPGSAGIGSEIPT-SSPLAP
      Acypi000094 TSSYPTRYDFIDIEAVMNNERIIKILFNCVMNQGPCTRE-GLELKRIVPDAIQTECAKCNDRQRKQAGKVLAHLLQYKPEYWNMLVKKFDPNNVYLRKYMADNDDDEKLSLQKLTNNTTK
        BonmoCSP4 TSTYTTQYDEVDIKEIMGNERLLVAYIGCLLDKNPCTPE-GKELKRNIPDALQSDCSKCSDKQRENADAWIEFMIDNRPEDWTKLEERYNPDGSYRTKYLEGKHNATSNVDESK
      Acypi000096 EEKYTTKFDNFDVDKVLNNDRILTSYIKCLLDQGNCTNE-GRELKKVLPDALKTDCSKCTAVQKDRSEKVIKFLIKNRSKDFDNLTAKYDPSGEYKKKIEKFDAERAAAAKH
      Acypi000095 QEKYSTKYENFDVDKVLNNDSLLTSYINCLLDEGNCTEE-GQALKRILPDALKTNCGKCTDAQKLKIEKIMKFLIKNRSIDFDRLTAKYDPSGEYKKKLEKFSA
      Pedhu594660 PQKYSTKYDNIDIDSILKNDRLLESHVNCLLDKGPCTKE-GQDFKDYLPEAIDTSCEKCTVAQKRIVKKAAKYLMEKKKPAWEQIRNKYDPMNEHTKDFNKFMEEPIE
      Acypi009116 PAKYTTKYDNVNIDDILNNDRLVASYFKCLMETGKCTPE-GEEIKRWLPEAVENKCEDCSEKQKIGSEKIIKFLFEKKNDMWKQLEEKYDPKGLYRQRYSEDAKKLNIDV
      Pedhu594430 DEKYSTKYDNIDLDSILKNDRLLQNYVNCLLDKGTCTPE-GTDLKKVLPDALENACAKCSEAQKRGAEKVIRHLLENKKDVFTLLEAKYDPNGVYRKKYEAEAQKRKIQL
      Pedhu594540 PTKFTTKFDDVNLDEILKSERLLKNYFDCLMDTKPCTPD-GTELKKSLPVALKEECAGCSEKQRVGAETVIHYISNNKPELWKALEDKYDPEQVYRTKHADFAAKKGIKV
       BonmoCSP14 ESTYTDKWDNINVDEILESNRLLKGYVDCLLGKGRCTPD-GKALKETLPDALEHECVKCTGKQKSGADKVIRHLVNKRPDLWKELAVKYDPDNIYQARYKDKIDAVKGSA
XP028028218Thap1 ESTYTDKWDNINVDEILESTRLMKGYVDCLLERGRCTPD-GKALKETLPDALEHECVKCTGKQKSGADKVIRHLVNKRPDLWKELAVKYDPDNIYQARYKDKIDAVKGSA
        BonmoCSP1 DDKYTDKYDKINLQEILENKRLLESYMDCVLGKGKCTPE-GKELKDHLQEALETGCEKCTEAQEKGAETSIDYLIKNELEIWKELTAHFDPDGKWRKKYEDRAKAKGIVIPE
        BonmoCSP8 DDKYTDRYDNVNLDEVLSNSRLLQPYIKCILDKDRCAPD-AKELKEHIREALETECAKCTEAQKKGTRRVIGHLINNESKSWNELTAKYDPENKFTAKYEKELREIKA
        BonmoCSP9 PEQYTDKYDTVDLDQLISNRRLLIPYVHCILEKGQCTAE-GKELKSHIKEALETNCAKCTKAQKGGTEKMIGHLINHEAEFWEELKAKYDPTNEFTKKYETELKRVTA
 BacamWP256881044 SSTYTDKYDNINIDEILSNKRLLTPYIKCMLDEIKCSTE-GKELKSHIKDALENECIKCTDAQKSGTRKVIGHLINKEPEYWSQLTKKYDPKQHYSQKYEKEL
XP028025219Thap1 AGSYSDRYDNINVAEILGNKRLLTAYIKCVLEEGKCTAE-GKELKSHITDALQTGCTKCTGAQRKGIRRVIKHLIDSEPGYWDRLVDMYDPKRVYTGKYEKELRTIKA
        BonmoCSP6 AEKYTDKYDNIDVDEILENRKLLVPYIKCVLDEGRCTPD-GKELKAHIKDGMQTACAKCTDKQKVSARKIVKHIKQHEADYWEQMKAKYDPKDEFKEIYEGFLAGQN
        BonmoCSP7 IARPKTPFDNINIEEIFENRRLLLGYINCILERGNCTRA-GKDLKSSLKNVLEENCDKCSEDQRKSIIKVINYLVSSEPESWNQLKSKYDPEGKYLIKYEAKMESN
      Pedhu594550 ISSYSTRYEHINLDDVLYNNRLLKNTYKCMIGTGRCTAE-ANKLKAYFPDMLETKCSKCTPRQKKKIKKAVTFLMKHKPEMWEGLLDKYDPKRELSKDMLTDPNFLGKI
       BonmoCSP15 AEFYSSRYDDFDVKPLVENDRILQSYTNCFLDKGPCTPD-AKEFKKVIPEALETTCGKCSPKQKQLIKTVIKAVIERHPEAWEELVNKYDKDRKFRPSFDKFINEDD
       BonmoCSP11 EEYYSSQYDNFDVEQLVGNLRLLKNYAKCFLDQGPCTAE-GTEFKKRIPEALRTKCAKCNPKQRHLIRTVVKAFQTKLPDLWEELAIKEDPKGQYKHEFTAFINAMD
XP028029186Thap1 EEYYSSQYDNFDVEQLVGNLRLLKNYAKCFLDQGPCTAE-GTEFKKRIPEALRTKCAKCNPKQRHLIRTVVKAFQTKLPDLWEELAIKEDPKGQYKQEFTAFINATD
       BonmoCSP13 DLFYDKKYDNFNVDEIIDNPRLLKAYTFCFNDKGKCTAE-GNDFKKWIPESLQTSCGKCSEKQKYLVAKFVHAIKDKMPDEFDILRKLHDPKGEYTENLDKFLETYGH
        BonmoCSP3 LAADLSKYENFDVEPIVTSDRLLKAYINCFLDKGRCTPE-ASDFKKALPDTIATNCGKCTEKQKANVRKVIKVIQQKHSTEWEKLVKKHDPSGKHRADFDKFLLGS
XP012549936Thap1 IWCYDEKYDKIDVDKILSDDKLFTDYINCMLDKGPCEVEYSSEFKELLPEVIATSCAKCTPIQKTGLRKTVKALSVKRPDDFSQFRAKYDPKGEYEKQFAAFVVATD
       BonmoCSP20 QKYYDSRYDYYDIDHLVQNPRLLKKYLDCFLGKGPCTPI-GRLFKQVMPEVITTACAKCTPTQKRFARKTFNAFRRYFPETLMELRRKFDPESKYYDAFEKVITNA
      Acypi002311 LKKFLSTLEKIDIDQILNNHRLMSNNVKCFLNEGPCTAQ-LREMKKMLPALVKDSCASCTKEQKNIIKKSMEAIQARRPNEYKQVSKFFDPEGKYQKKFLENLNTD
       BonmoCSP17 RQQSYPRNDNININAILQNDRILLGYFKCVMDRGPCTKD-GKTFKRALPEALPTACARCSNKQKAAFRTLLLAIRARSEPSFLELLDKYDPSRSNRELLYTFLATGL
XP030028674Thap1 TQQNYPRNENININAILQNDRILLGYYKCVMDKGPCTKD-GKVFKRALPEALPTACARCSQKQKAVFRTLLLAIRAKSEPRFMELLDKYDPSRANRDTLYKFLATGV
         OIC81003 QDKYEPIDDSFDASEVLSNERLLKSYTKCLLNQGPCTAE-LKKIKDKIPEALETHCAKCTDKQKQMAKQLAQGIKKTHPELWDEFITFYDPEGKYQTSFKDFLES
        BonmoCSP2 QDKYEPIDDSFDASEVLSNERLLKSYTKCLLNQGPCTAE-LKKIKDKIPEALETHCAKCTDKQKQMAKQLAQGIKKTHPELWDEFITFYDPQGKYQTSFKDFLES
         OIC85870 QDKYEPIDDSFDASEVLSNERLLKSYTKCLLNQGPCTAE-LKKIKDKIPEALETHCAKCTDKQKQMAKQLAQ
      Acypi000345 SGYYLSTYDNLDVGHLLRNKKVVSGFVKCFVNEGPCTPDGKLVKAYLLPEIIRTVCGKCTPRQKDMSRAVLRHLYTYRRADFDKIMQIYDTDNKKNEIINFMNQK
       BonmoCSP12 KETYSSENDDLDIEALVGNIDSLKAFIGCFLETSPCDAV-SGDFKKDIPEAVAEACGKCTPAQKHLFKRFLEVVKDKLPQEYEAFKTKYDPQGKHFDALLSAVANS
 KlepnWP159189367 GEHYTDRYDNVDLDEILSNKKVLENYVKCILDQGKCTPD-GTELKGHIKDALETECAKCNERQKDGTRRHHGEHYT
      Acypi005842 KDSSLPNVSEDVLDKALSDRRFVQRQLKCATGEGPCDPI-GRKIKAHAPLVLRGMCVKCSQSEIKQIQRVMSHIQKNYPKEYTKMLKQYQSGF
       BonmoCSP19 AQQNRPQVTDTALDEALNDKRFIQRQLKCALGEAPCDPI-GKRLKTLAPLVLRGACPQCSPQETKQIQKTLSYVQRNFPQHWAKLVRQYAG
      Acypi003368 EDDDKPDFITTFDLIRLLDEKYAMKQFYCVINEDPCDSV-GMRLKATIPEEINRDCERCTATETNNIRRILNYVKKHYPKFWERVEPIYRDKMTVLTANIVKIQ
       BonmoCSP16                                              AHITDALQTGCTKCTGAQRKGIRRVIKHLIDSEPGYWDRLVDMYDPKRVYTGKYEKELRTIKA
       BonmoCSP18                                               LLPEVIATSCAKCTPIQKTGLRKTVKALSVKRPDDFSQFRAKYDPKGEYEKQFAAFVVATD
        BonmoCSP5 AGSYSDRYDNINVAEILGNKRLLTAYIKCVLEEGKCTAE-GKELK
        Consensus ...y...yd..d.d..l.n.rll..y..C.l..gpCt.e g..lk...p.al.t.C.kCt..#k....k....l....p..w..l..kydp...y.................

                  131  140       150       160       170       180       190       200       210       220       230       240       250       260
                  |--------+---------+---------+---------+---------+---------+---------+---------+---------+---------+---------+---------+---------|
             Mp10
      Acypi000097
       BonmoCSP10 PPPPSRPASVFTNPPPPVMSTSPRPVVLNRYLFNNLFFIKEDRRTCSQRETQMKLKVR
        HelzePAN1 PRAPTVAPTVATPTPAATEPTPPRPVVLNRFGDEGELMMGSPSSAGITPRPMTQATTRPTTTMRPVNTRPVPPRPTMMTWAGAASNTQPTRFPLRPSPSDVPPPYSTAITLIDQIGYKIIKTTELVTDLL
      Acypi000094
        BonmoCSP4
      Acypi000096
      Acypi000095
      Pedhu594660
      Acypi009116
      Pedhu594430
      Pedhu594540
       BonmoCSP14
XP028028218Thap1
        BonmoCSP1
        BonmoCSP8
        BonmoCSP9
 BacamWP256881044
XP028025219Thap1
        BonmoCSP6
        BonmoCSP7
      Pedhu594550
       BonmoCSP15
       BonmoCSP11
XP028029186Thap1
       BonmoCSP13
        BonmoCSP3
XP012549936Thap1
       BonmoCSP20
      Acypi002311
       BonmoCSP17
XP030028674Thap1
         OIC81003
        BonmoCSP2
         OIC85870
      Acypi000345
       BonmoCSP12
 KlepnWP159189367
      Acypi005842
       BonmoCSP19
      Acypi003368
       BonmoCSP16
       BonmoCSP18
        BonmoCSP5
        Consensus

                  261    270
                  |--------|
             Mp10
      Acypi000097
       BonmoCSP10
        HelzePAN1 RNTVRAVVGR
      Acypi000094
        BonmoCSP4
      Acypi000096
      Acypi000095
      Pedhu594660
      Acypi009116
      Pedhu594430
      Pedhu594540
       BonmoCSP14
XP028028218Thap1
        BonmoCSP1
        BonmoCSP8
        BonmoCSP9
 BacamWP256881044
XP028025219Thap1
        BonmoCSP6
        BonmoCSP7
      Pedhu594550
       BonmoCSP15
       BonmoCSP11
XP028029186Thap1
       BonmoCSP13
        BonmoCSP3
XP012549936Thap1
       BonmoCSP20
      Acypi002311
       BonmoCSP17
XP030028674Thap1
         OIC81003
        BonmoCSP2
         OIC85870
      Acypi000345
       BonmoCSP12
 KlepnWP159189367
      Acypi005842
       BonmoCSP19
      Acypi003368
       BonmoCSP16
       BonmoCSP18
        BonmoCSP5
        Consensus
```
