# Supplementary material for: Renaming the ‘OS-D/CSP’ Family (Part 1): ‘4-Cysteine Soluble Proteins’ (4CSPs)—Molecular Nomenclature, Structure, Expression, Evolution, Tissue-Distribution, and Pleiotropy
Source: Insects. 2026 Feb 13;17(2):202. doi: 10.3390/insects17020202 (PMC12940638; doi:10.3390/insects17020202)
Supplement: Supplementary file 1 [file insects-17-00202-s001.zip › Liuetal.Insects2025-Part1-TableS1.pdf]

**Organism**	**Order**	**Genome Assembly**	**Gene Structure**	**Transcript Annotation**	**Length (bps)**	**N-terminus**	**C-terminus**	**Identity % (Order subject)**
Acyrthosiphon pisum *Acypi*	Hemiptera	EQ110797	E1-8697-E2	Acypi000094	1236	TSSYPTRY	KLTNNTTK	92 (Hemiptera) 42 (Enterobacterales)
		EQ110797	E1-1283-E2	Acypi009116	363	PAKYTTKY	AKKLNIDV	90 (Hemiptera) 59 (Myxococcales)*
		EQ116326	E1-3103-E2	Acypi002311	1039	LKKFLSTL	FLENLNTD	78 (Hemiptera) 35 (Lysobacterales)
		EQ117790	E1-586-E2	Acypi000097	815	GTAYTTKY	KKKGVVKF	97 (Hemiptera) 75 (Enterobacterales)
		EQ121783	E1-1378-E2	Acypi000093	812	FPAYMKRF	KLNKFLDA	90 (Hemiptera) 43 (Bacillales)
		EQ121783	E1-4739-E2	Acypi000345	1554	SGYYLSTY	IINFMNQK	80 (Hemiptera) 47 (Lysobacterales)
		EQ122410	E1-698-E2-16718-E3	Acypi005842	844	KDSSLPNV	LKQYQSGF	96 (Hemiptera) 29 (Kitasatosporales)
		EQ125317	E1-735-E2	Acypi000096	669	FTLAEEKY	ERAAAAKH	95 (Hemiptera) 56 (Enterobacterales)
		EQ125317	E1-3103-E2	Acypi003368	2009	EDDDKPDF	TANIVKIQ	79 (Hemiptera) 30 (Bacillales)
		EQ126525	E1-272-E2	Acypi000095	645	QEKYSTKY	KKLEKFSA	91 (Hemiptera) 58 (Enterobacterales)
Bombyx mori	Lepidoptera	BABH0	E1-93-E2-359-E3	Bommo-CSP19	351	AQQNRPQV	AKLRQYAG	88 (Lepidoptera)

Bommo		1005933		Bommo-4CSP19				80 (Kitasatosporales)
		BABH01018301	Pseudogene (E2)	Bommo-CSP16 Bommo-4CSP16	189	--------	KELRTIKA	98 Allergen Thap1† 59 (Bacillales)
		BABH01021421	E1-693-E2-880-E3	Bommo-CSP4 Bommo-4CSP4	624	TSTYYTTQ	TSNVDESK	56 (Lepidoptera) 81 (Kitasatosporales)
		BABH01021423	E1-2020-E2	Bommo-CSP9 Bommo-4CSP9	375	PEQYTDKY	TELKRVTA	64 Allergen Thap1† 58 (Bacillales)
		BABH01021424	E1-747-E2	Bommo-CSP6 Bommo-4CSP6	393	AEKYTDKY	EGFLAGQN	70 (Lepidoptera) 61 (Lysobacterales)
		BABH01021424	E1-776-E2	Bommo-CSP7 Bommo-4CSP7	366	IARPKTPF	YEAKMESN	51 (Lepidoptera) 91 (Kitasatosporales)
		BABH01021424	E1-566-E2	Bommo-CSP8 Bommo-4CSP8	384	DDKYTDRY	ELDREIKA	77 (Lepidoptera) 73 (Enterobacterales)
		BABH01021425	E1-580-E2	Bommo-CSP1 Bommo-4CSP1	504	DDKYTDKY	AKGIVIPE	76 Allergen Thap1† 57 (Myxococcales)*
		BABH01021426	Intronless	Bommo-CSP14 Bommo-4CSP14	360	ESTYTDKW	IDAVKGSA	96 Allergen Thap1† 57 (Lysobacterales)
		BABH01021426	E1-803-E2	Bommo-CSP15 Bommo-4CSP15	366	AEFYSSRY	DKFINEDD	75 Lepidoptera 51 (Enterobacterales)
		BABH01021427	E1-2703-E2	Bommo-CSP2 Bommo-4CSP2	360	QDKYEPID	SFKDFLES	68 (Lepidoptera) 56 (Bacillales)
		BABH0	E1-5137-E2	Bommo-CSP17	372	RQQSYPRN	YTFLATGL	84 Allergen

		102142<br>9		Bommo-4CSP17				Thap1†<br>46<br>(Lysobacterales)
		BABH0<br>102143<br>0	E1-768-E2	Bommo-CSP3<br>Bommo-4CSP3	754	LAADLSKY	ADKFLLGS	72 (Lepidoptera)<br>47<br>(Lysobacterales)
		BABH0<br>102143<br>0	Pseudogene (E2)	Bommo-CSP18<br>Bommo-4CSP18	123	--------	AAFVVATD	100 Allergen<br>Thap1†<br>46 (Bacillales)
		BABH0<br>102143<br>1	E1-3718-E2	Bommo-CSP20<br>Bommo-4CSP20	402	QKYYDSRY	FEKVITNA	69 (Lepidoptera)<br>46<br>(Myxococcales)*
		BABH0<br>102143<br>3	E1-403-E2	Bommo-CSP12<br>Bommo-4CSP12	366	KETYSSEN	LLSAVANS	66 (Lepidoptera)<br>39<br>(Lysobacterales)
		BABH0<br>102143<br>4	E1-843-E2	Bommo-CSP11<br>Bommo-4CSP11	372	EEYYSSQY	TAFINAMD	98 Allergen<br>Thap1†<br>50<br>(Lysobacterales)
		BABH0<br>102146<br>7	E1-2171-E2	Bommo-CSP13<br>Bommo-4CSP13	381	DLFYDKKY	KFLETYGH	61 (Lepidoptera)<br>54<br>(Myxococcales)*
		BABH0<br>102170<br>9	E1-26820-E2-<br>2758-E3	Bommo-CSP10<br>Bommo-4CSP10	1798	MPKYDERY	TQMKLKVR	87 PAN1-like√<br>47<br>(Alteromodales)**
		BABH0<br>103464<br>2	Pseudogene (E1)	Bommo-CSP5<br>Bommo-4CSP5	123	AGSYSDRY	--------	100 Allergen<br>Thap1†<br>68 (Bacillales)

Pediculus humanus humanus *Pedhu*	Phthiraptera	AAZ00 100724 1	E1-187-E2-100-E3	Pedhu594410	429	GEKYTTRW	NDNKENSR	68 (Diptera) 63 (Lysobacterales)
		AAZ00 100724 1	E1-288-E2	Pedhu594420	399	PTKFTTKF	AAQKGITV	55 (Diptera) 56 (Lysobacterales)
		AAZ00 100724 1	E1-277-E2	Pedhu594430	451	DEKYSTKY	AQKRKIQL	58 (Diptera) 61 (Lysobacterales)
		AAZ00 100724 2	E1-208-E2	Pedhu594540	423	PTKFTTKF	DFAAKIKV	54 (Diptera) 49 (Lysobacterales)
		AAZ00 100724 3	E1-89-E2	Pedhu594550	384	ISSYSTRY	DPNFLGKI	44 (Hymenoptera) 42 (Bacillales)
		AAZ00 100724 3	E1-143-E2	Pedhu594660	348	PQKYSTKY	KFMEEPIE	46 (Coleoptera) 57 (Myxococcales)*

Table S1. Aphid '4CSP' gene repertoire in comparison to that of moths and lice, genome assembly, gene structure, and protein identity. *Acypi*: *Acyrthosiphon pisum*, pea aphid (Hemiptera, Aphididae); *Bommo*: *Bombyx mori*, domestic mulberry silkworm moth (Lepidoptera, Bombycidae); *Pedhu*: *Pediculus humanus humanus*, body louse (Psocodea, Phthiraptera, Pediculidae).
Thap1: *Thaumetopoea pityocampa* (Pine processionary moth, Lepidoptera, Notodontidae) isoallergen and variant (following Allergen Nomenclature: 15-kDa IgE-binding protein). † Expression of Thap1 in silkgland (XP_028029186).
√PAN-1: Protein (cytoplasmic and transmembrane domains), encoded by the pan-1 gene (necessary for developmental processes). Gene truncations are indicated by the dashed lines (pseudogenes).
In Gene Structure, E: Exon, E1: Exon1, E2: Exon2, E3: Exon3. The size of the intron is indicated by the interval between exons.
Outcomes from the Microbial NCBI database using Blastp (Blast®, Microbes, Microbial Protein BLAST).
* *Sorangiinae bacterium* MSr11954. ** *Shewanella electrica*.

Bommos is now known as "4-Cysteine Soluble Proteins" ("4CSPs") instead of "Chemosensory Proteins" ("CSPs").
