# Supplementary figures and images for: Renaming the ‘OS-D/CSP’ Family (Part 1): ‘4-Cysteine Soluble Proteins’ (4CSPs)—Molecular Nomenclature, Structure, Expression, Evolution, Tissue-Distribution, and Pleiotropy
Source: Insects. 2026 Feb 13;17(2):202. doi: 10.3390/insects17020202 (PMC12940638; doi:10.3390/insects17020202)

**A.**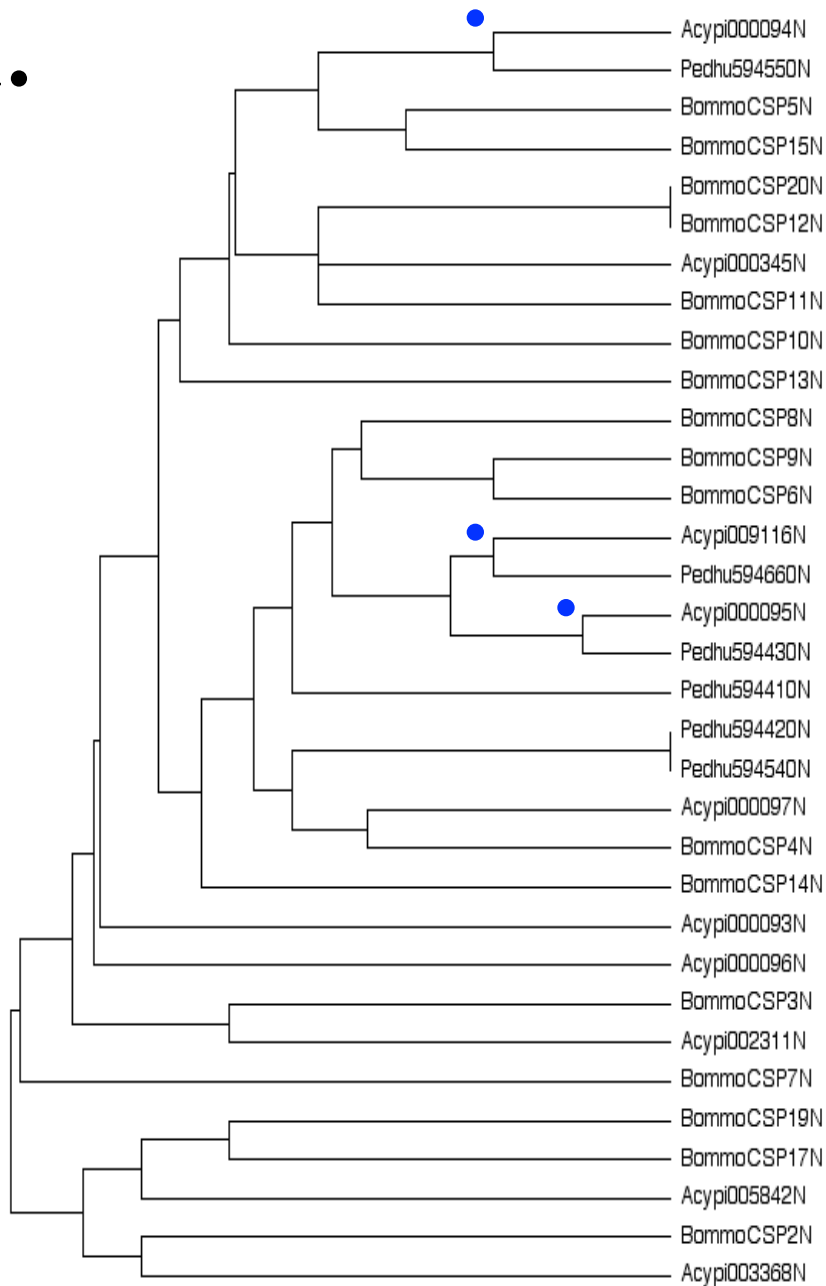

0.1 changes

**B.**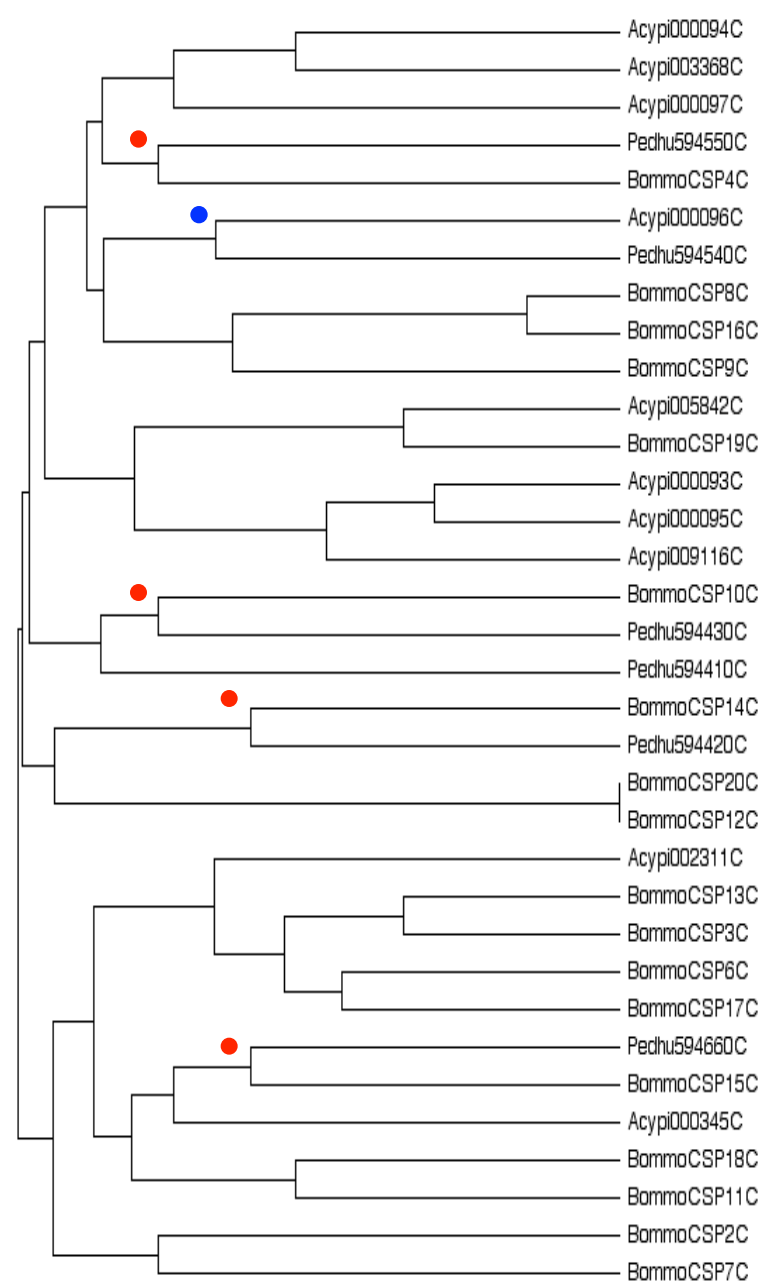

0.1 changes

Supplement: Supplementary file 1 [file insects-17-00202-s001.zip › Liuetal.Insects2025-Part1-FigureS1.pdf]

**A.**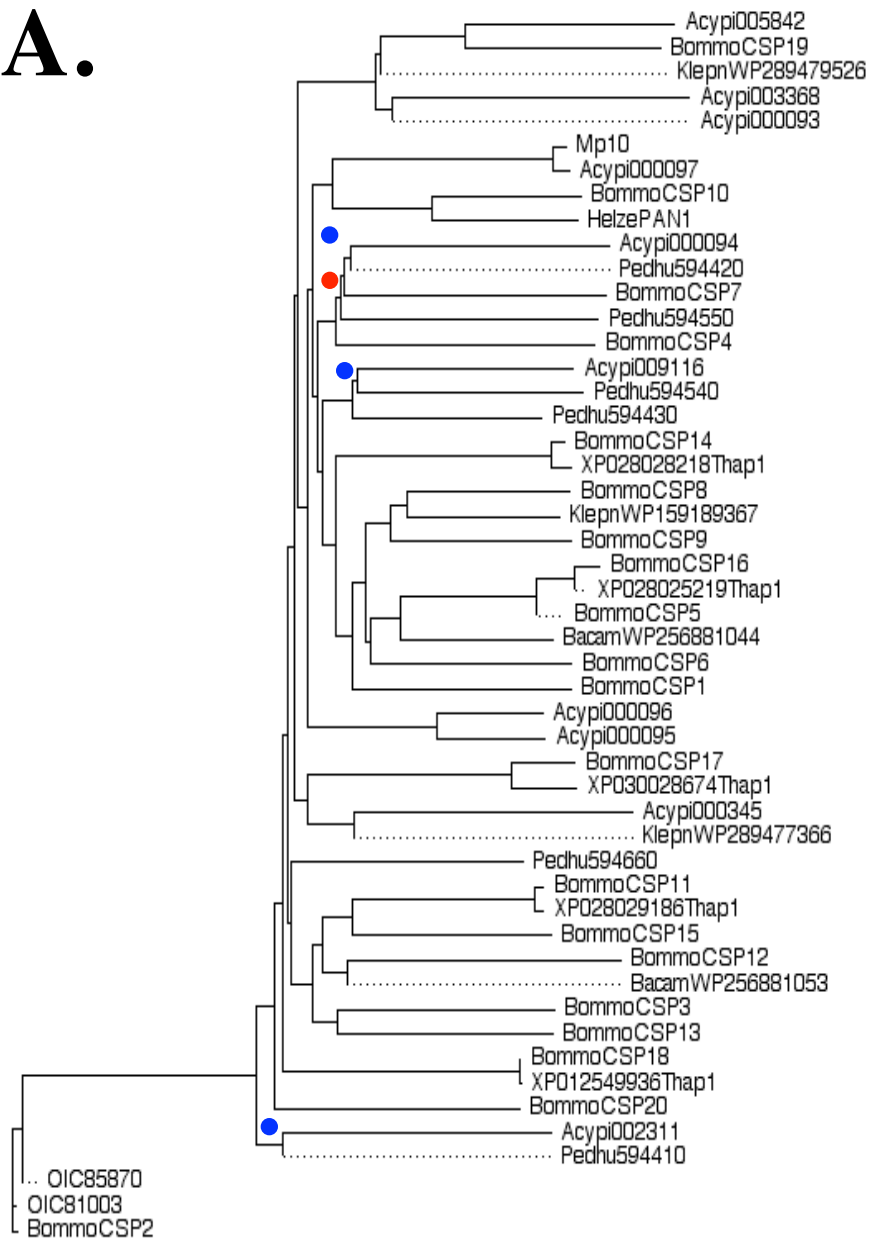

0.05 changes

**B.**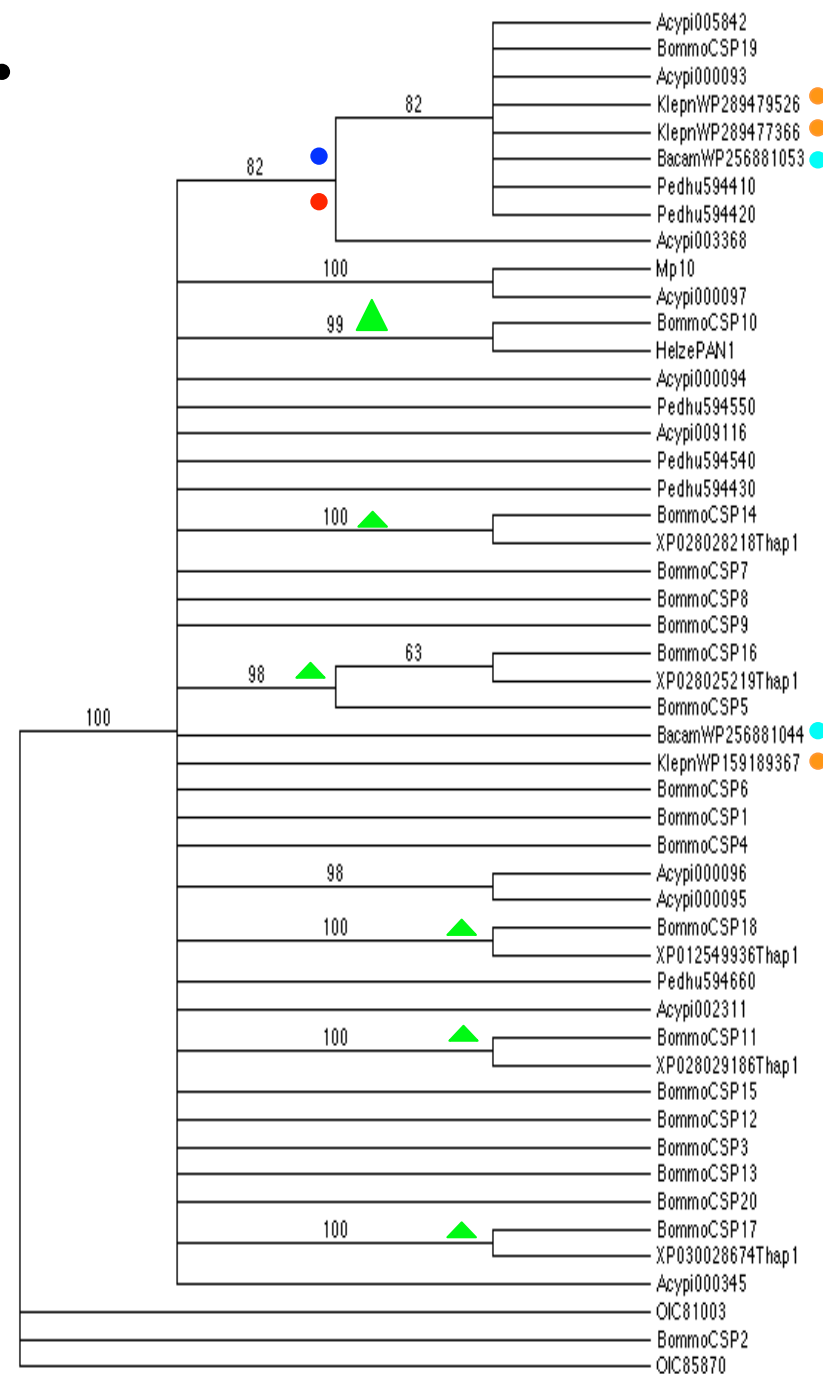

Supplement: Supplementary file 1 [file insects-17-00202-s001.zip › Liuetal.Insects2025-Part1-FigureS2.pdf]

# A.

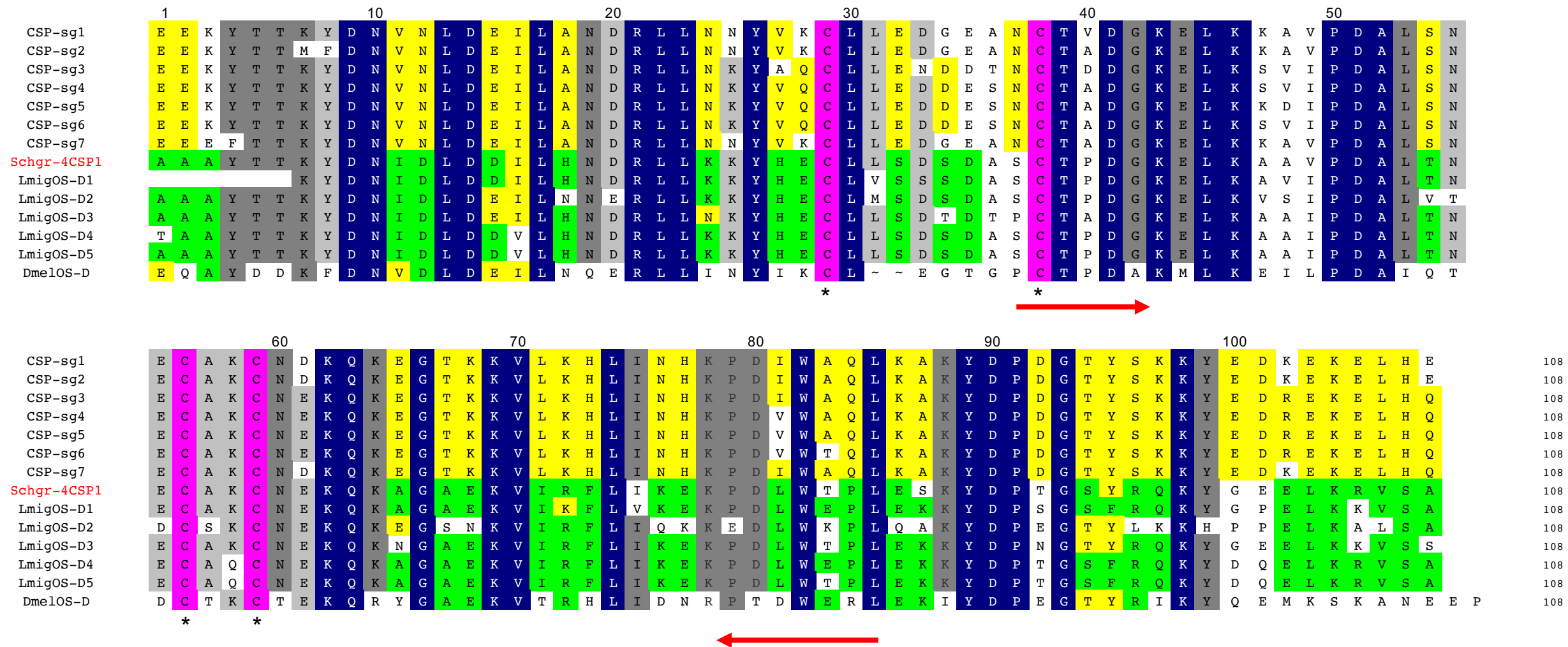

B.

4<sup>th</sup> instar larvae

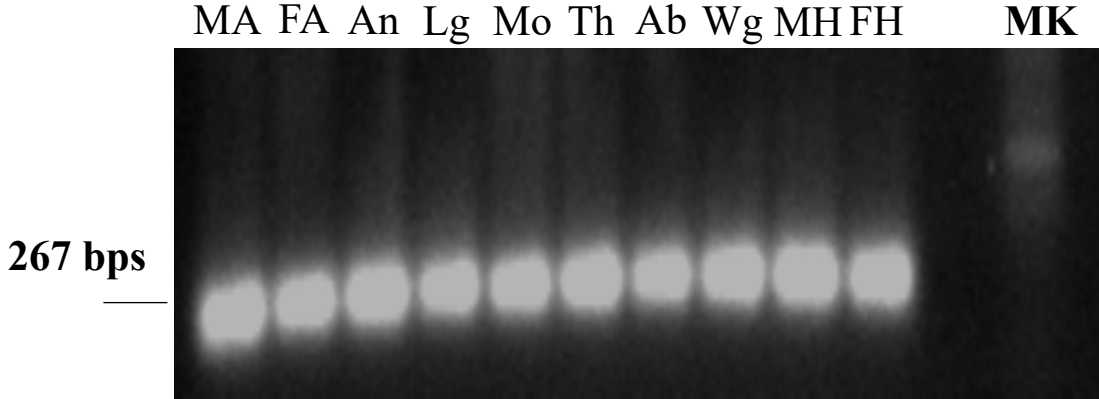

16SrRNA

4<sup>th</sup> instar larvae

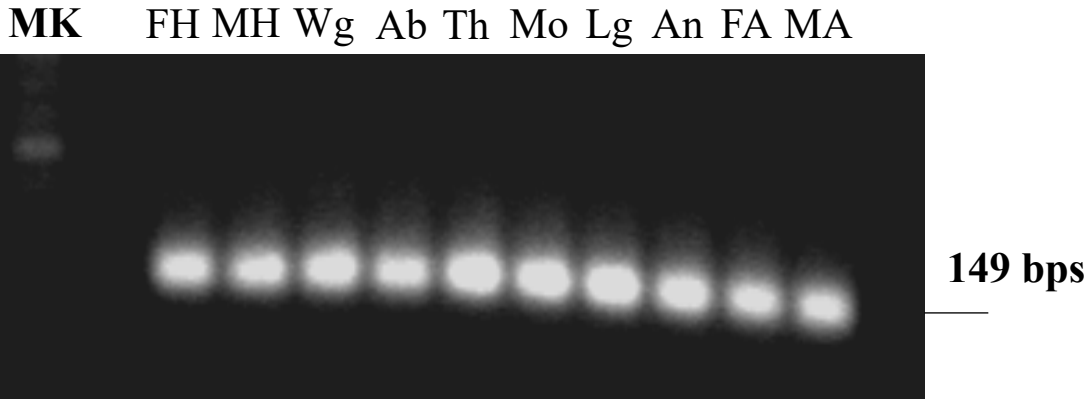

Schgr-4CSP1

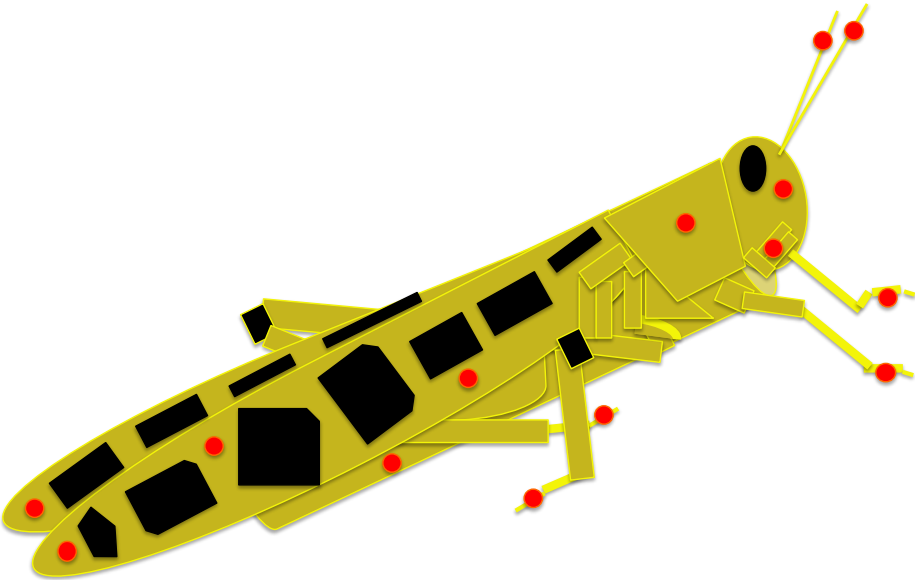

Supplement: Supplementary file 1 [file insects-17-00202-s001.zip › Liuetal.Insects2025-Part1-FigureS4 .pdf]

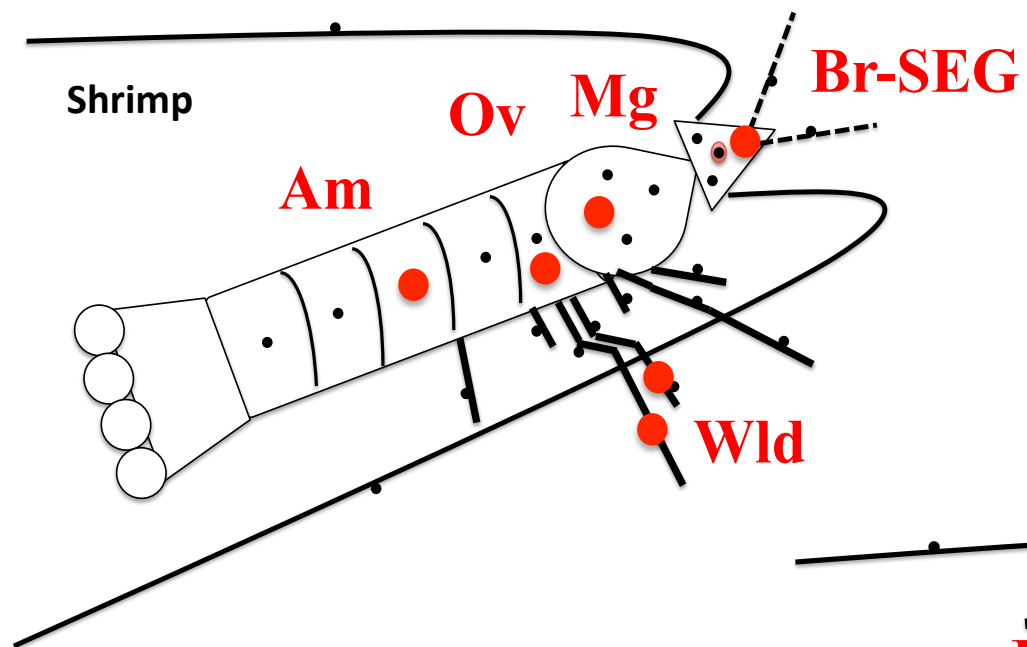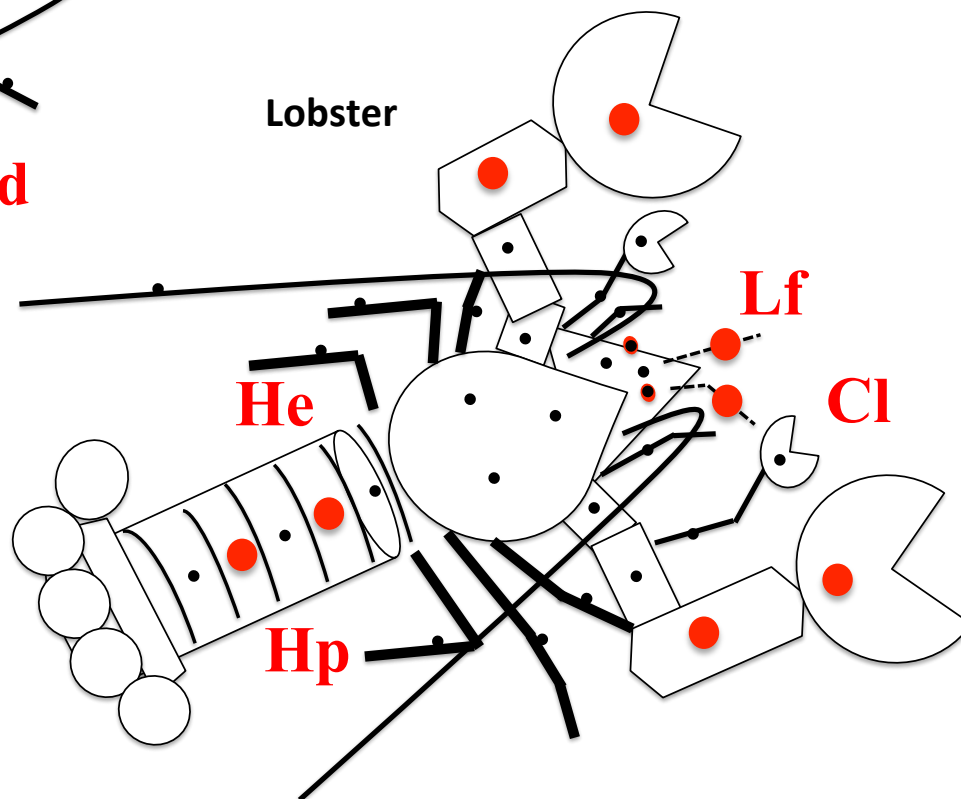

Supplement: Supplementary file 1 [file insects-17-00202-s001.zip › Liuetal.Insects2025-Part1-FigureS5.pdf]
